# Disordered region of cereblon is required for efficient degradation by proteolysis-targeting chimera

**DOI:** 10.1038/s41598-019-56177-5

**Published:** 2019-12-23

**Authors:** Kidae Kim, Dong Ho Lee, Sungryul Park, Seung-Hyun Jo, Bonsu Ku, Sung Goo Park, Byoung Chul Park, Yeong Uk Jeon, Sunjoo Ahn, Chung Hyo Kang, Daehee Hwang, Sehyun Chae, Jae Du Ha, Sunhong Kim, Jong Yeon Hwang, Jeong-Hoon Kim

**Affiliations:** 1 Disease Target Structure Research Center, Korea Research Institute of Bioscience and Biotechnology (KRIBB), Daejeon, 34141 Republic of Korea; 2 Department of Proteome Structural biology, KRIBB School of Bioscience, Korea University of Science and Technology, Daejeon, 34113 Republic of Korea; 3 Therapeutics & Biotechnology, Korea Research Institute of Chemical Technology, Daejeon, 34114 Republic of Korea; 4 Department of Functional Genomics, KRIBB School of Bioscience, Korea University of Science and Technology, Daejeon, 34113 Republic of Korea; 5 Department of Medicinal Chemistry and Pharmacology, Korea University of Science and Technology, Daejeon, 34113 Republic of Korea; 6 Bio & Drug Discovery Division, Korea Research Institute of Chemical Technology, Daejeon, 34114 Republic of Korea; 7 College of Pharmacy, Chungnam National University, Daejeon, 34134 Republic of Korea; 8 Department of Biological Sciences, Seoul National University, Seoul, 08826 Republic of Korea; 9 Korea Brain Bank, Korea Brain Research Institute, Daegu, 41062 Republic of Korea; 10 Department of Bio-Molecular Science, KRIBB School of Bioscience, Korea University of Science and Technology, Daejeon, 34113 Republic of Korea

**Keywords:** Proteasome, Mechanism of action

## Abstract

Proteolysis targeting chimeras (PROTACs) are an emerging strategy for promoting targeted protein degradation by inducing the proximity between targeted proteins and E3 ubiquitin ligases. Although successful degradation of numerous proteins by PROTACs has been demonstrated, the elements that determine the degradability of PROTAC-targeted proteins have not yet been explored. In this study, we developed von Hippel-Lindau-Cereblon (VHL-CRBN) heterodimerizing PROTACs that induce the degradation of CRBN, but not VHL. A quantitative proteomic analysis further revealed that VHL-CRBN heterodimerizing PROTACs induced the degradation of CRBN, but not the well-known immunomodulatory drug (IMiD) neo-substrates, IKAROS family zinc finger 1 (IKZF1) and −3 (IZKF3). Moreover, truncation of disordered regions of CRBN and the androgen receptor (AR) attenuated their PROTAC-induced degradation, and attachment of the disordered region to stable CRBN or AR facilitated PROTAC-induced degradation. Thus, these results suggest that the intrinsically disordered region of targeted proteins is essential for efficient proteolysis, providing a novel criterion for choosing degradable protein targets.

## Introduction

Targeted protein degradation is a rapidly emerging therapeutic intervention that induces protein degradation rather than causing catalytic inhibition. Proteolysis-targeting chimeras (PROTACs)—the first reported protein degraders—are hetero-bifunctional compounds that contain a linker and two ligands: one recruits an E3 ubiquitin ligase, and the other binds to the target protein^1^. PROTACs promote formation of a ternary complex with the target protein and the E3 ligase, leading to ubiquitination and subsequent proteasomal degradation of the target protein^2–4^. Although many therapeutic target proteins have been identified, a majority of these proteins, which include transcription factors, scaffold proteins and non-enzymatic proteins, are known to be difficult to modulate by small molecules. However, PROTACs can target these undruggable proteins through ubiquitin proteasome system (UPS)-dependent protein destruction^5,6^. A number of target proteins, including nuclear, cytosolic and membrane proteins, have subsequently been successfully eliminated by PROTACs using E3 binders for von Hippel-Lindau (VHL), cereblon (CRBN), beta-transducin-repeats–containing protein (β-TRCP), and mouse double minute 2 homolog (MDM2)^1,7–11^. The most widely used E3 binders are VHL ligands, which bind to VHL, and immunomodulatory drugs (IMiDs) that recognize CRBN. Crews and colleagues, inspired by the crystal structure of the hydroxylated hypoxia-inducible factor 1-alpha (HIF1α) peptide bound to the Cullin2-RING ubiquitin Ligase (CRL2^VHL^) complex^12^, designed VHL ligands as inhibitors of VHL and HIF1α protein-protein interactions.

IMiDs are thalidomide analogues containing a glutarimide moiety that have anti-myeloma, anti-inflammatory and anti-proliferative effects^13–15^. The cellular target of IMiDs had not been discovered until Ito and colleagues showed that the receptor for IMiDs is CRBN, which forms a CRL4^CRBN^ complex with damage-specific DNA-binding protein 1 (DDB1), Cullin4 (CUL4), and RBX1^16^. Once IMiDs bind to CRBN, CRL4^CRBN^ induces degradation of CxxCG-containing Zinc finger proteins, such as IKZF1, IKZF3, ZFP91, SALL4 and ZNF827; it also degrades casein kinase 1 alpha (CK1α), but only in the case of lenalidomide^17–19^. Several endogenous CRBN substrates, including the homeobox protein Meis2 (MEIS2), C-JUN and the glutamine synthetase (GLUL), have been identified^20–22^. For example, CRBN recognizes two acetylated lysine residues at the N-terminal region of GLUL and eliminates unnecessary acetylated GLUL under conditions of high levels of glutamine^22^. Apart from its E3 ligase activity, CRBN possesses pleotropic functions. It directly inhibits activation of AMPK by interacting with the α1 subunit of AMP-activated protein kinase (AMPK α1) and plays a role in maturation of basigin (BSG) and solute carrier family 16 member 1 (SLC16A1) in a ubiquitin-independent manner^23,24^.

It has been demonstrated that the disordered region in proteins is associated with proteasome-mediated degradation. In addition to the polyubiquitin chain, efficient proteasomal degradation requires that a substrate have a terminally or internally located unfolded or disordered region. Once a substrate binds to the proteasome, this disordered region can act as the degradation initiation site^25–29^. Therefore, globular proteins that lack a disordered region are inefficiently degraded, even when polyubiquitinated. Alternatively, such globular proteins can be unfolded by the p97/valosin-containing protein (VCP) complex in an ATP-dependent manner and then directly degraded by the proteasome^30^. The relationship between intrinsically disordered regions of targeted proteins and PROTAC-mediated proteolysis, however, remains unknown.

Two previous studies demonstrated that VHL-CRBN heterodimerizing PROTACs promote CRBN degradation, but not VHL degradation, and that treatment with these PROTACs causes resistance of multiple myeloma cells to IMiDs^31,32^. Here, using an unbiased proteome analysis, we show that our VHL-CRBN heterodimerizing PROTACs also induce efficient degradation of CRBN. CRBN degradation by these PROTACs recapitulates a CRBN deficiency in cells. We further propose that the intrinsically disordered region of targeted proteins is required for efficient proteolysis by PROTACs.

## Results

### CRBN is efficiently degraded by VHL-CRBN heterodimerizing PROTACs

**Figure 1:**
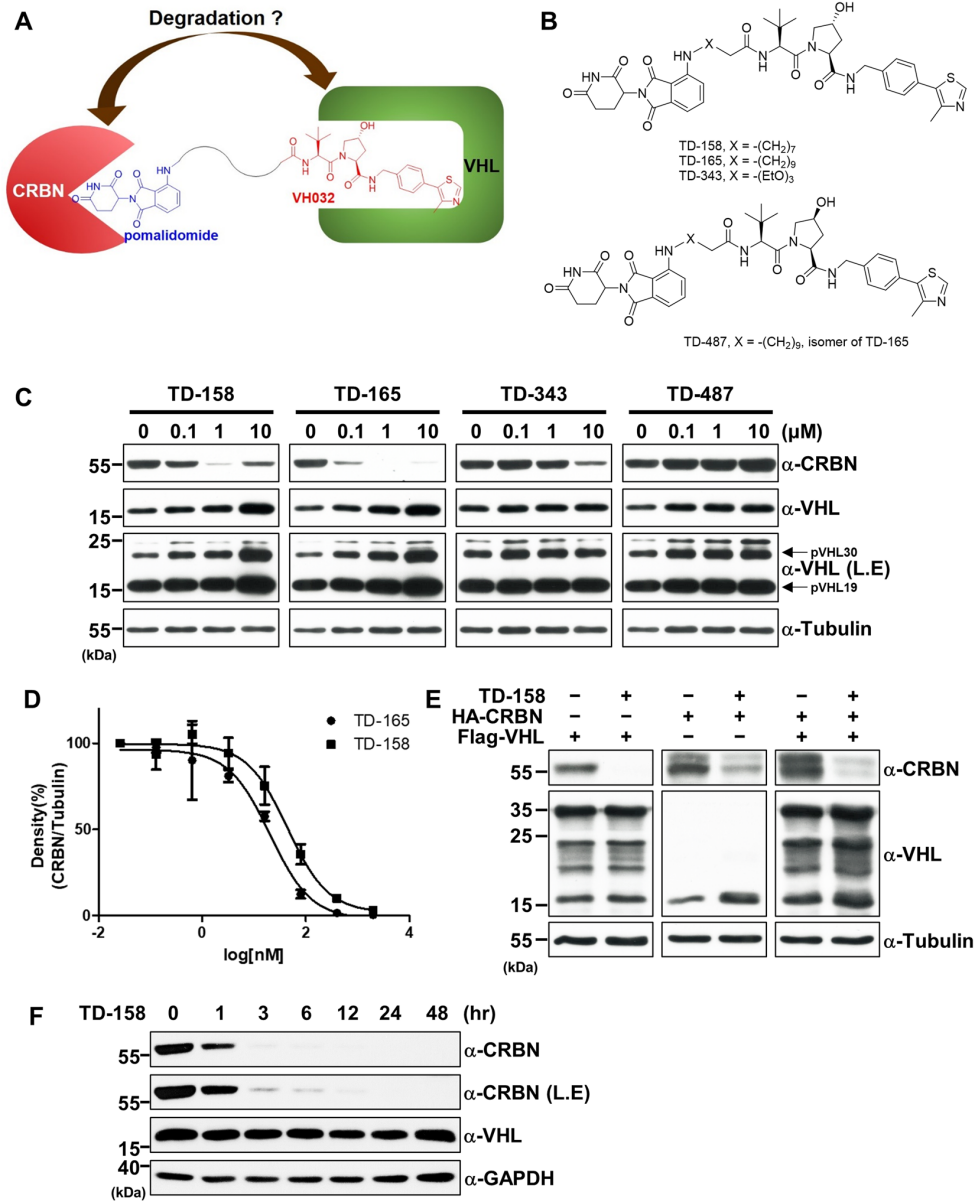
CRBN is efficiently degraded by VHL-CRBN heterodimerizing PROTACs. (**A**) Schematic diagram of the PROTAC containing pomalidomide and the VHL ligand (VH032). (**B**) Structure of TD-158, TD-165, TD-343, and TD-487. (**C**) HEK293T cells were treated with TD-158, TD-165, TD-343 or TD-487 (0.1, 1, and 10 μM) for 24 h, and VHL protein levels were analyzed by immunoblotting. L.E. indicates long exposure of the Western blot. (**D**) DC_50_ graph of TD-158 and TD-165 compounds (TD- 165, DC_50_ = 20.4 nM; TD-158, DC_50_ = 44.5 nM). (**E**) HA-CRBN and Flag-VHL were expressed in HEK293T cells. After 24 h, the cells were treated with TD-158 (500 nM) for 24 h. Whole cell lysates were analyzed by immunoblotting for the indicated proteins. (**F**) HEK293T cells were treated with 500 nM TD-158 at different time points, and CRBN levels were analyzed by immunoblotting. L.E. indicates long exposure of the Western blot.

PROTAC technology utilizes E3 ligases to destroy target proteins. We thus wondered whether an E3 ligase itself can be ubiquitinated and degraded by another E3 ligase if two different E3 ligases are placed in proximity. VHL degraders potentially might be erythropoietin substitutes through activation of HIF1α. To design this, we connected pomalidomide, a CRBN-targeting molecule, to VHL032, a VHL E3 ligase ligand (Fig. 1A). The linker connected the amino group of pomalidomide and the terminal acetyl group of VH032; because these are solvent-exposed regions, a connection between them would not likely perturb their binding to each E3 ligase. For the synthesis of VHL-CRBN heterodimerizing PROTACs (Fig. 1B and Supplementary Fig. S1A), 4-fluorothalidomide (**1**) and VHL ligand (**5**) were prepared as previously reported^33^. 4-Fluorothalidomide (**1**) was treated with four amine linkers (**2**) bearing a *tert*-butyl ester group in the presence of N,N-diisopropylethylamine (DIPEA) in dimethyl sulfoxide (DMSO) to form an intermediate (**3**). Following deprotection of the *tert*-butyl group in **3** by treatment with trifluoroacetic acid (TFA) in dichloromethane (DCM), the acid (**4**) was coupled with the VHL ligand (**5** or **7**) in the presence of 1-[bis(dimethylamino)methylene]−1H-1,2,3-triazolo[4,5-b]pyridinium 3-oxide hexafluorophosphate (HATU) and DIPEA to yield the PROTACS (**6**), TD-158, TD-165, TD-343 and TD-487 (**8**).

After treatment with these compounds, we examined the levels of VHL and CRBN by Western blot analysis. Treatment with TD-158, TD-165, or TD-343 caused a concentration-dependent decrease in the level of CRBN proteins (Fig. 1C, upper panel). Levels of both VHL long form and VHL short form, however, increased, presumably owing to stabilization by the chemical-protein interaction (Fig. 1C, upper-middle and lower-middle panels)^34^. In the case of treatment with 10 μM TD-158, CRBN levels were restored; this “hook” effect is a typical characteristic that occurs in the context of high-dose PROTAC-induced protein degradation^9,35–37^. However, TD-487, a stereoisomer of TD-165, failed to induce CRBN degradation (Fig. 1C). TD-343 possessed relatively weak activity, implying that incorporation of oxygen in the linker inhibits PROTAC activity. A quantitative reverse transcription-polymerase chain reaction (RT-PCR) analysis indicated that the decrease in CRBN level induced by VHL-CRBN heterodimerizing PROTACs did not result from changes in the mRNA (Supplementary Fig. S1B). The 50% degradation concentration (DC_50_) and maximum degradation (D_max_) values were measured for all synthesized compounds. The calculated DC_50_ and D_max_ values for TD-158 were 44.5 nM and 97.1%, respectively, and the corresponding values for TD-165 were 20.4 nM and 99.6% (Fig. 1D and Supplementary Fig. S1D). The shorter linker-containing degrader TD-156 (DC_50_ = 100.6 nM, D_max_ = 96.9%) displayed weaker activity compared with TD-165 (Supplementary Fig. S1C). TD-343 (DC_50_ = 367.8 nM, D_max_ = 85.1%), with oxygen incorporated in the linker, led to inefficient degradation of CRBN. To test the effect of the linkage to thalidomide, we examined degradation of CRBN by VHL-CRBN heterodimerizing PROTACs with a different linkage. TD-760 (DC_50_ = 367.7 nM, D_max_ = 82%), with a glycolic linkage, and TD-033 (DC_50_ = 2546.1 nM), with an amide linkage, exhibited reduced CRBN degradation, whereas TD-759 (DC_50_ = 28.8 nM), with a glycine linkage, displayed a potency similar to that of TD-165. To determine whether relative protein levels might contribute to this biased protein degradation, we treated cells overexpressing VHL, CRBN, or both with TD-158, and analyzed CRBN and VHL levels. In all cases, CRBN levels were reduced while VHL levels remained similar or increased (Fig. 1E). In time-course experiments, TD-158 degraded CRBN by more than 80% within 3 h and completely degraded it after 12 h (Fig. 1F). TD-158-induced CRBN degradation was also observed in various human cell lines (Supplementary Fig. S2A–D).

### Degradation of CRBN by VHL-CRBN heterodimerizing PROTACs is dependent on CRL2^VHL^

**Figure 2:**
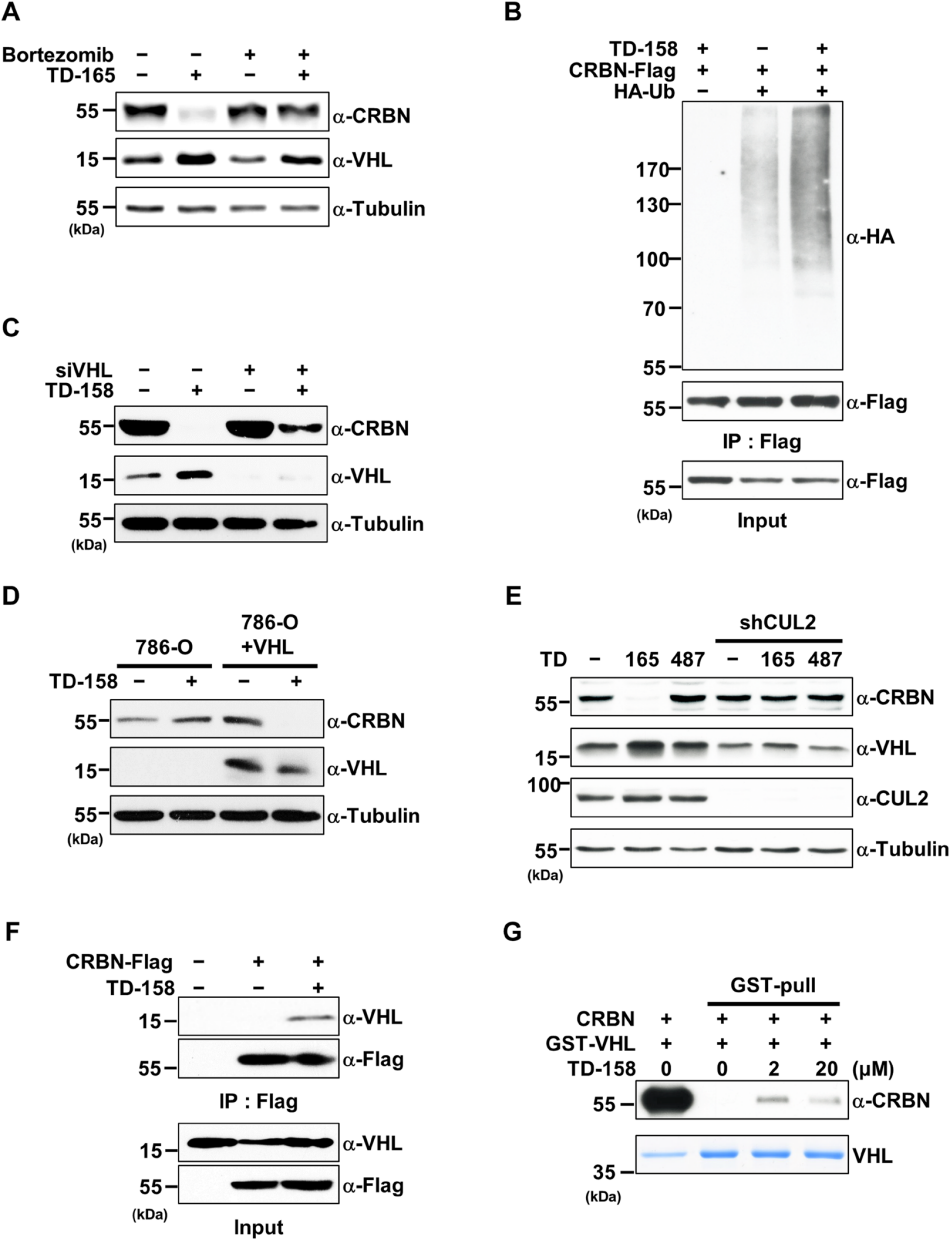
Degradation of CRBN by VHL-CRBN heterodimerizing PROTACs is dependent on CRL2^VHL^. (**A**) HEK293T cells were treated with TD-165 (1 μM) for 12 h, followed by addition of bortezomib (20 nM) or DMSO for 8 h. Whole-cell lysates were subjected to immunoblot analysis for the indicated proteins. (**B**) FLAG-tagged CRBN (CRBN-Flag) and HA-tagged Ubiquitin (HA-Ub) were expressed in HEK293T cells. After 24 h, the cells were treated with TD-158 (500 nM) and bortezomib (20 nM), or DMSO and bortezomib (20 nM), for 12 h. Whole-cell lysates and proteins immunoprecipitated using Flag M2 magnetic beads were analyzed by immunoblotting for the indicated proteins. (**C**) HEK293T cells were transfected with siRNA specific for VHL or scrambled (control) siRNA. After 24 h, the cells were treated with TD-158 (500 nM) for 24 h. Whole-cell lysates were analyzed by immunoblotting for the indicated proteins. (**D**) 786-O cells and VHL-expressing 786-O cells (786-O + VHL) were treated with TD-158 (500 nM) for 24 h. Whole-cell lysates were analyzed by immunoblotting for the indicated proteins. (**E**) HEK293T and CUL2- knockdowned HEK293T cells were treated with TD-165 (1 μM) or TD-487 (1 μM) for 24 h and whole-cell lysates were analyzed by immunoblotting. (**F**) CRBN-Flag was expressed in HEK293T cells. After 36 h, the cells were treated with TD-158 (1 μM) and bortezomib (20 nM), or DMSO and bortezomib (20 nM), for 12 h. Whole-cell lysates and proteins immunoprecipitated using Flag M2 magnetic beads were analyzed by immunoblotting for the indicated proteins. (**G**) Purified CRBN and VHL/ELOB/ELOC complex proteins were mixed and aliquoted into four tubes. TD-158 and glutathione beads were added as indicated and incubated for 3 h. After incubation, glutathione beads were washed and analyzed by immunoblotting and Coomassie Blue staining.

To verify that CRBN degradation by TD-158 was dependent on UPS or Cullin-RING ubiquitin ligases (CRL), we tested bortezomib, a proteasome inhibitor, and MLN4924, a neddylation inhibitor. Co-treatment with TD-158 and bortezomib or MLN4924 prevented CRBN degradation (Fig. 2A and Supplementary Fig. S3A). As expected, poly-ubiquitin chain formation increased markedly in the presence of TD-158 (Fig. 2B). In addition, knockdown of VHL by small interfering RNA (siRNA) attenuated TD-158–induced CRBN degradation in HEK293T cells (Fig. 2C). In contrast, expression of VHL in 786-O cells, a VHL-deficient renal carcinoma cell line, restored TD-158–induced CRBN degradation (Fig. 2D). In line with these results, siRNA-mediated knockdown of Cullin2, a scaffolding protein of the CRL2^VHL^ complex, prevented TD-158–induced CRBN degradation (Fig. 2E). Collectively, these results indicate that CRBN degradation by VHL-CRBN heterodimerizing PROTACs is dependent on the CRL2^VHL^ complex.

Efficient degradation by PROTACs requires formation of a ternary complex with the target protein and E3 ligase^9,38^. To test this, we performed co-immunoprecipitation experiments. We found that exogenously expressed Flag-tagged CRBN co-immunoprecipitated endogenous VHL in the presence of TD-158, but not in its absence (Fig. 2F). GST pull-down experiments using purified proteins showed that CRBN directly interacted with the VHL-Elongin B/C complex in the presence of TD-158 (Fig. 2G). Moreover, the addition of excess pomalidomide or VHL ligand inhibited TD-158–induced CRBN degradation (Supplementary Fig. S3B,C). Exogenously expressed CRBN Y384/W386A (AA), a pomalidomide-binding–defective mutant^16^, was not degraded by TD-158 (Supplementary Fig. S3D). Thus, these data suggest that formation of a ternary complex among CRBN, VHL, and TD-158 is a prerequisite for CRBN degradation.

### A global proteomic analysis reveals that TD-158 induces degradation of CRBN

**Figure 3:**
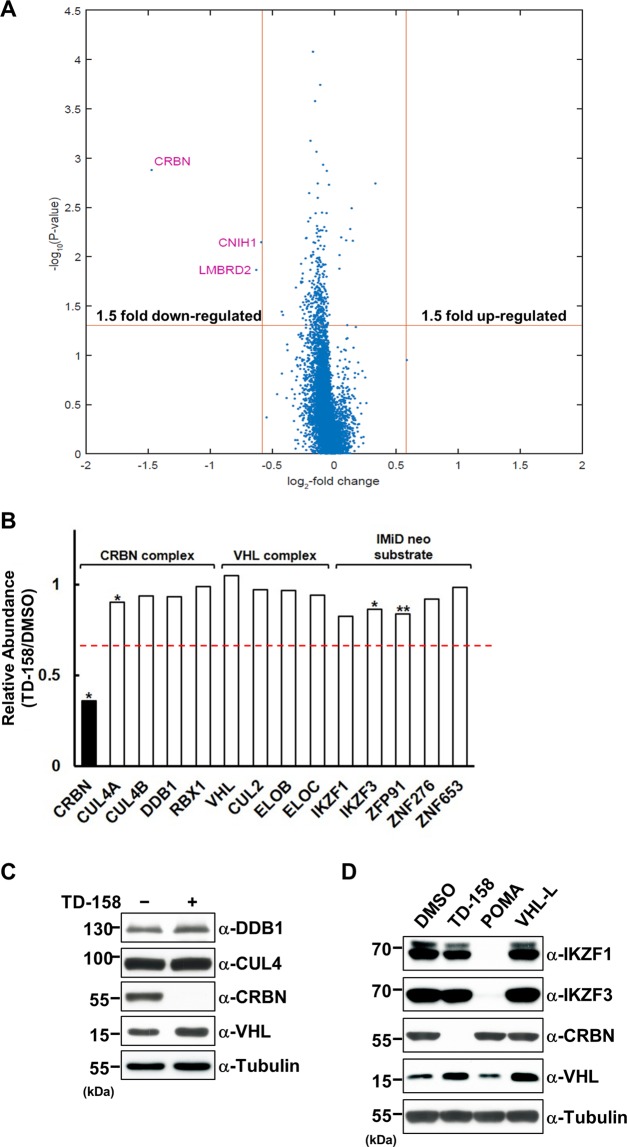
Global proteomic changes upon TD-158 treatment. (**A**) Volcano plot of proteins identified by quantitative proteomic analyses, comparing lysates from Jurkat cells treated for 12 h with 1 μM TD-158 or DMSO. Data represent three biological replicates. The x-axis represents the fold-change in protein levels in logarithmic scale, and the y-axis represents *P*-value in logarithmic scale. (**B**) Relative abundance of CRL4^CRBN^ complex, CRL2^VHL^ complex, and neo-substrate of CRBN (*P < 0.05, **P < 0.1). The red line indicates a 1.5-fold difference. (**C**) Jurkat cells were treated with TD-158 (1 μM) or DMSO for 24 h. Whole cell lysates were analyzed by immunoblotting for CRL4^CRBN^ complex proteins. (**D**) Jurkat cells were treated with or without DMSO, TD-158 (1 μM), pomalidomide (1 μM), or VHL ligand (1 μM) for 24 h. Whole-cell lysates were analyzed by immunoblotting for the indicated proteins.

To examine the degradation of CRBN in an unbiased fashion, we performed a quantitative proteomic analysis. To minimize the secondary effects of CRBN degradation, we treated Jurkat cells, which expressed CRBN and IMiD neo substrates, with TD-158 or DMSO (vehicle control) for 12 h. The proteins from each sample were digested and the digested peptides were tagged for isobaric labeling coupled to liquid chromatography-tandem mass spectrometry. This analysis identified 7,148 proteins containing more than three non-redundant, unique peptides (Supplementary Table S1). Only three proteins—CRBN, CNIH1 and LMBRD2—met the criteria of a P-value = 0.05 and more than a 1.5-fold change. Among them, CRBN was the only protein that changed by more than 2-fold (Fig. 3A). However, levels of other components of CRL4^CRBN^ complexes (CUL4A, CUL4B, DDB1, and RBX1) and CRL2^VHL^ complexes (elongin C [ELOC], elongin B [ELOB], CUL2, and RBX1) remained unchanged (Fig. 3B,C). Interestingly, the levels of previously reported neo-substrates of IMiDs, including IKZF1, IKZF3, ZFP91, ZNF276 and ZNF653, were not changed upon TD-158 treatment (Fig. 3B)^39–41^. These results were confirmed by immunoblotting in Jurkat and various multiple myeloma cell lines (Fig. 3D and Supplementary Fig. S2A–D).

### CRBN degradation by VHL-CRBN heterodimerizing PROTACs recapitulates a CRBN deficiency

**Figure 4:**
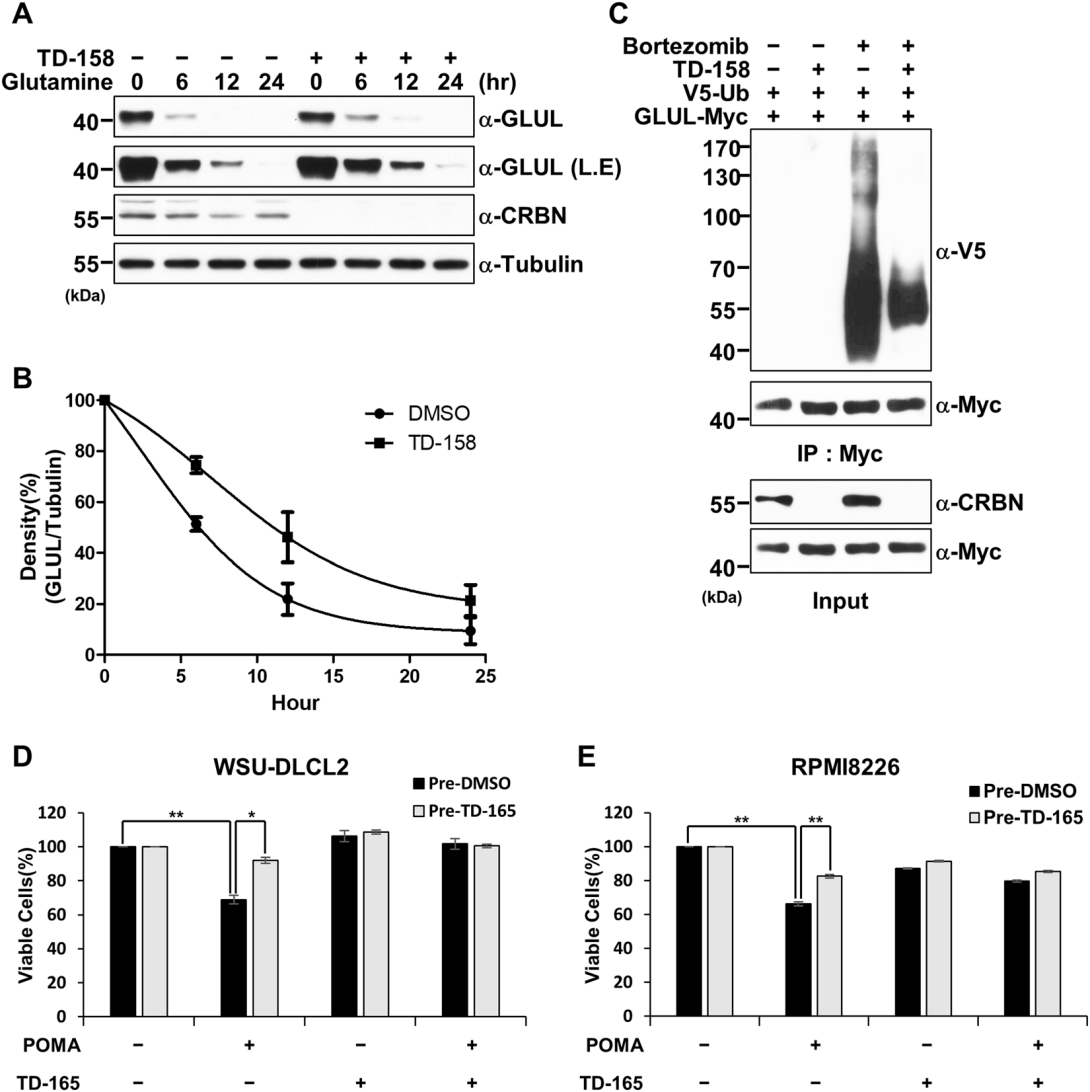
CRBN degradation by VHL-CRBN heterodimerizing PROTACs recapitulates a CRBN deficiency. (**A**) Hep3B cells were glutamine-starved and treated with TD-158 (500 nM) for 48 h. The cells were then treated with glutamine (4 mM) at different time points. Degradation of GLUL was analyzed by immunoblotting. (**B**) Quantitative results from three independent experiments. (**C**) GLUL-Myc and V5-Ub were expressed in HEK293T cells. After 24 h, the cells were treated with TD-158 (500 nM) or DMSO for 12 h and then treated with bortezomib (100 nM) or DMSO for 12 h. Whole-cell lysates and proteins immunoprecipitated using Myc magnetic beads were analyzed by immunoblotting for the indicated proteins. (**D,E**) WSU-DLCL2 (**D**) and RPMI8226 (**E**) cells were pre-treated with TD-165 (1 μM) or DMSO for 24 h and, after harvesting, were divided into four groups. Each group was then treated with pomalidomide (1 μM) and DMSO, or pomalidomide (1 μM) and TD-165 (1 μM), for 3 d. Cell viability was measured using CellTiter-Glo (**P < 0.001, *P < 0.01).

An endogenous substrate of CRL4^CRBN^ is the glutamine synthetase GLUL, which is a key enzyme in the biogenesis of glutamine. The acetyltransferase CBP/p300 acetylates two N-terminal lysine residues of GLUL under conditions of high levels of glutamine, and the resulting acetylated GLUL is captured and ubiquitinated by CRL4^CRBN^ and subsequently removed by proteasomes^22^. To examine the effect of TD-158 on GLUL levels, we starved Hep3B cells of glutamine for 48 h and then resupplied glutamine in the presence or absence of TD-158. TD-158 induced CRBN degradation regardless of glutamine status, and the levels of GLUL decreased more slowly over time in the presence of TD-158 than in its absence (Fig. 4A,B). Moreover, *in vivo* ubiquitination assays showed that ubiquitination of GLUL decreased in the presence of TD-158 (Fig. 4C). We also investigated whether TD-165 treatment confers cellular resistance to IMiD. Two IMiD-sensitive cell lines, WSU-DLCL2 and RPMI8226, were pre-treated with TD-165 or DMSO for 24 h and then treated with pomalidomide, TD-165, or both for 3 d. Pre-treatment with TD-165 reduced the anti-proliferative effects of pomalidomide in both cell lines (Fig. 4D,E). Taken together, these data indicate that CRBN degradation by VHL-CRBN heterodimerizing PROTACs recapitulates a CRBN deficiency.

To investigate the *in vivo* effects of VHL-CRBN heterodimerizing PROTACs, we attempted to determine whether TD-165 can induce CRBN degradation in animal models. Although amino acid residues in mouse CRBN (mCRBN) important for teratogenicity are not conserved, mCRBN was distinctly degraded, albeit to a slightly lesser extent, by TD-165 in mouse embryonic fibroblasts (Supplementary Fig. S4A). We then administered TD-165 intraperitoneally to mice to determine whether TD-165 induces CRBN degradation *in vivo*. However, CRBN levels were not changed in the spleen, peripheral blood mononuclear cells (PBMCs), or liver (Supplementary Fig. S4B). Given that the pharmacokinetics of TD-165 are reasonable (Supplementary Table S2), this absence of an effect might be attributable to the high plasma protein binding (99.9%) of TD-165 (Supplementary Table S3).

### N-terminally truncated CRBN is not degraded by VHL-CRBN heterodimerizing PROTACs

**Figure 5:**
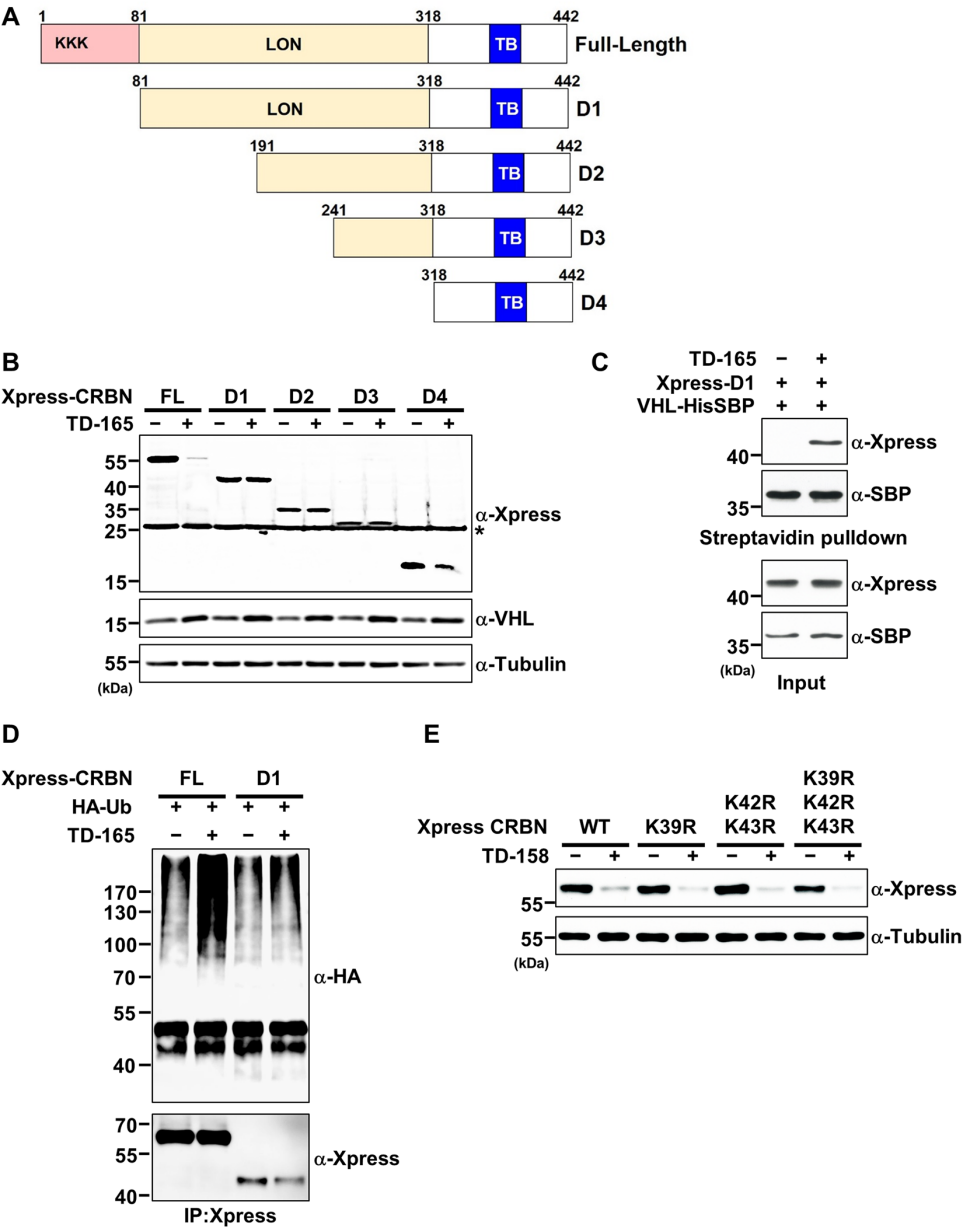
The N-terminal disordered region of CRBN is necessary for degradation, but not for ubiquitination, by VHL-CRBN heterodimerizing PROTACs. (**A**) Schematic diagram illustrating CRBN truncation mutants. LON, Lon protease domain; TB, thalidomide binding domain. (**B**) Xpress-tagged full-length CRBN or D1, D2, D3, or D4 deletion mutants were expressed in HEK293T cells. After 24 h, the cells were treated with TD-165 (3 μM) or DMSO for 24 h. Whole-cell lysates were analyzed by immunoblotting for the indicated proteins. (**C**) Plasmids encoding Xpress-tagged D1 and His-SBP–tagged VHL were transfected into HEK293T cells. After 48 h, cells were treated with TD-165 (1 μM) or DMSO for 24 h. Whole-cell lysates and proteins pull-downed using streptavidin beads were analyzed by immunoblotting for the indicated proteins. (**D**) Plasmids encoding Xpress-tagged CRBN, K39R, K42/43 R, or K39/42/43 R mutants were transfected into HEK293T cells. After 24 h, the cells were treated with TD-158 (2 μM) or DMSO for 24 h. Whole-cell lysates were analyzed by immunoblotting for the indicated proteins.

To determine which domain of CRBN is important for TD-165–mediated CRBN degradation, we generated a series of CRBN deletion mutants (D1–D4), as depicted in Fig. 5A. Cells expressing full-length CRBN or individual deletion mutants were treated with either DMSO or TD-165, and the cell lysates were examined for degradation after TD-165 treatment. Surprisingly, none of the four CRBN deletion mutants were degraded by TD-165, whereas full-length CRBN was efficiently degraded (Fig. 5B). Notably, VHL levels were slightly elevated presumably due to the stabilization of chemical-protein interaction in the presence of TD-165, but not altered by expression of truncated CRBN mutants in the presence of TD-165 (Fig. 5B). One possible explanation for this would be the inability of deletion mutants to form a ternary complex. However, the D1 mutant did form a ternary complex with TD-165 and VHL (Fig. 5C), and ubiquitination of D1 increased in the presence of TD-165 (Fig. 5D). We then hypothesized that amino-terminal lysine residues of CRBN are required for ubiquitination. To test this idea, we substituted all three lysine residues within the N-terminus of CRBN (a.a. 1–80) with arginine, individually and in combination, and then examined cell lysates for CRBN degradation. TD-158 efficiently degraded all mutants to the same extent as wild-type CRBN (Fig. 5E), indicating that the three lysine residues at the N-terminus of CRBN are not required for degradation induced by VHL-CRBN heterodimerizing PROTACs.

### The disordered region of the targeted protein is required for efficient PROTAC-induced degradation

**Figure 6:**
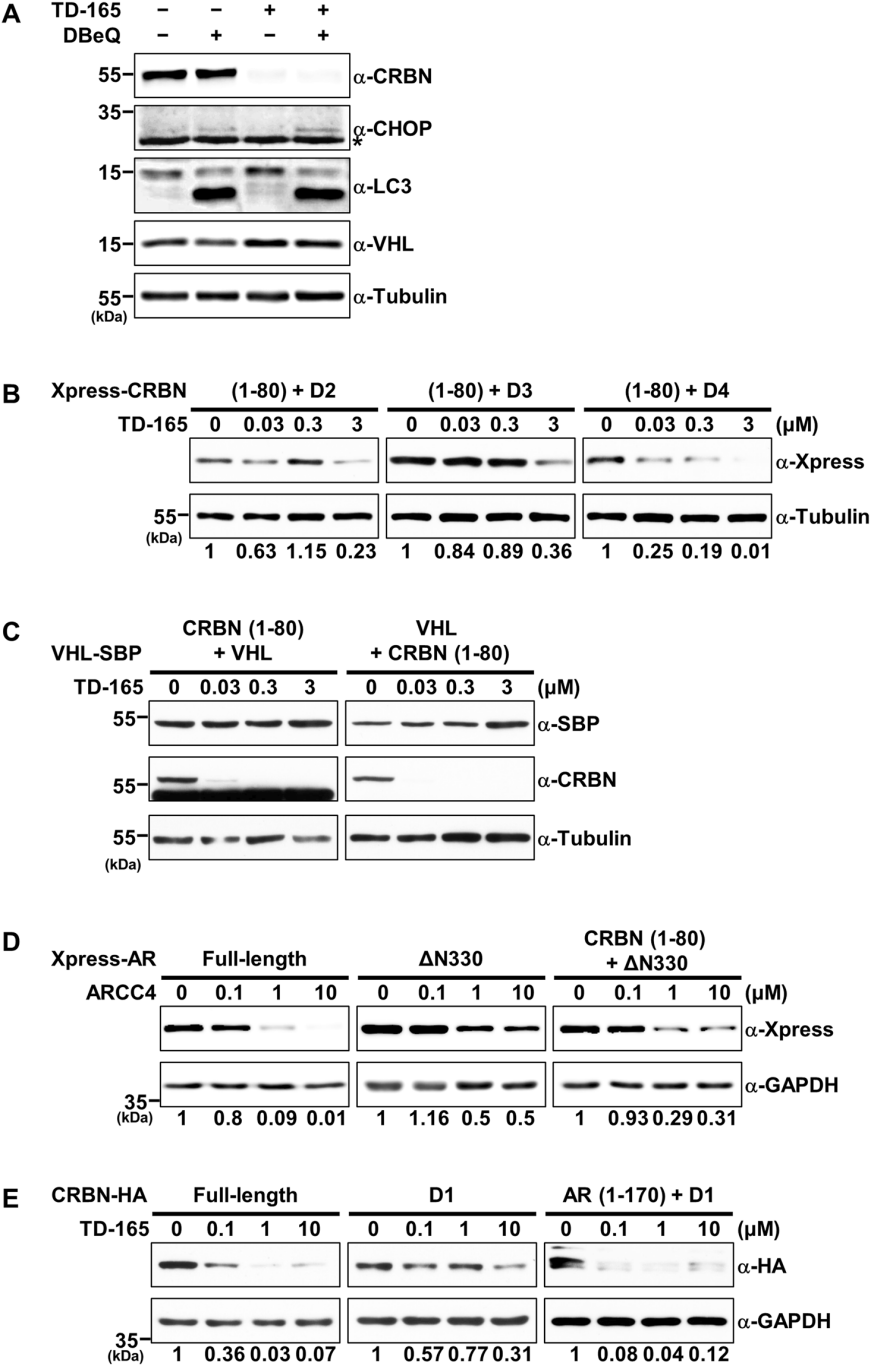
The disordered region of the targeted protein is required for efficient degradation by PROTACs. (**A**) HEK293T cells were treated with TD-165 (1 μM), the p97/VCP inhibitor DBeQ (10 μM), or both for 6 h. Whole-cell lysates were analyzed by immunoblotting for the indicated proteins. (**B**) N-terminal CRBN (a.a. 1–80) was inserted into the N- or C-terminus of His-SBP–tagged VHL plasmid, and plasmids were expressed in HEK293T cells. After 8 h, the cells were harvested and divided into four groups. Each group was then treated with increasing concentrations of TD-165 for 48 h. Whole-cell lysates were analyzed by immunoblotting for the indicated proteins. (**C**) Plasmids expressing the N-terminus of CRBN (a.a. 1–80) fused to D2, D3, or D4 were transfected into HEK293T cells. After 8 h, the cells were harvested and divided into four groups. Each group was then treated with increasing concentrations of TD-165 for 48 h. Whole-cell lysates were analyzed by immunoblotting for the indicated proteins. (**D**) Plasmids expressing full-length AR, N-terminally deleted AR (ΔN330), or the N-terminus of CRBN (a.a. 1–80) fused to ΔN330 (CRBN (a.a. 1–80) +ΔN330) were transfected into HEK293T cells. After 8 h, the cells were harvested and divided into four groups. Each group was then treated with increasing concentrations of ARCC4 for 24 h. Whole-cell lysates were analyzed by immunoblotting for the indicated proteins. (**E**) Plasmids expressing full-length CRBN, D1 deletion mutant, or N-terminus of AR (a.a. 1–170) fused to D1 (AR (1–170) +D1) were transfected into HEK293T cells. After 8 h, the cells were harvested and divided into four groups. Each group was then treated with increasing concentrations of TD-165 for 48 h. Whole-cell lysates were analyzed by immunoblotting for the indicated proteins.

We next sought to determine structural differences between full-length CRBN and the CRBN deletion mutant D1. The N-terminus of CRBN (a.a. 1–80) was predicted to contain an unfolded or intrinsically disordered region, based on an IUPred2A analysis (Supplementary Fig. S5A). This disordered region (a.a. 1–48) was indeed indiscernible in the CRL4^CRBN^ crystal structure owing to its flexibility (PDB entry; 6BN7). It has been demonstrated that the intrinsically disordered region is one of the main components of the proteasomal degron and it acts as the initiator for proteasomal proteolysis^25^. Alternatively, in the case of globular proteins without a disordered region, the p97/valosin-containing protein (p97/VCP) complex may unfold the secondary structure, facilitating the proteins’ proteasomal degradation. To examine the relationship between PROTAC-induced protein degradation and the p97/VCP complex, we tested the effect of N^2^,N^4^-dibenzylquinazoline-2,4-diamine (DBeQ), a p97/VCP inhibitor, on TD-165–induced CRBN degradation. These experiments showed the CRBN degradation was not influenced by DBeQ treatment (Fig. 6A). The levels of DNA-damage-inducible transcript 3 (DDIT-3; CHOP) and lipidated LC-3II, used as positive controls, were elevated upon DBeQ treatment. Knockdown of p97 by siRNA or DBeQ treatment impaired ERAD and autophagy pathways, leading to upregulation of CHOP, a well-established UPR marker, and accumulation of LC-3II, a representative autophagy marker, respectively (Fig. 6A)^42^. Therefore, to investigate whether the disordered region of CRBN facilitates PROTAC-induced proteasomal degradation, we attached the disordered region (a.a. 1–80) of CRBN to D2, D3, and D4 mutants and examined the chimeric proteins for degradation. All chimeric proteins, especially the D4 chimera, were degraded by TD-165 in a concentration-dependent manner (Fig. 6B). However, introduction of a CRBN disordered region to either the N- or C-terminus of VHL did not induce degradation of the fusion protein (Fig. 6C), indicating that attachment of the disordered region is not sufficient for all targeted proteins. To extend this idea to degradation induced by other PROTACs, we tested ARCC4, a previously reported androgen receptor (AR) PROTAC^43^, because AR also harbors an unfolded region at the N-terminus (a.a. 1–330), as determined using the IUPred2A analysis tool (Supplementary Fig. S5B). Upon ARCC4 treatment, AR without the N-terminal disordered region (ΔN330) was degraded less efficiently than the full-length AR. Intriguingly, attachment of the CRBN disordered region (a.a. 1–80) to AR ΔN330 increased the degradation efficiency (Fig. 6D and Supplementary Fig. S5C). In keeping with this, attachment of the AR disordered region (a.a. 1–170) to CRBN D1 mutant also promoted proteolysis by TD-165 (Fig. 6E and Supplementary Fig. S5D).

To further reinforce these findings, we selected PROTACs with high potency (DC_50_ < 1 μM) based on a literature search, and then analyzed them for an intrinsically disordered region using the IUPred2A tool. Interestingly, two-thirds of the selected PROTAC targeted proteins were found to possess a disordered region of more than 40 amino acids, either at one of their termini or internally (Table 1)^44^, although amino acid sequence-based prediction of disordered or unstructured regions does not take other factors, such as protein-protein interaction or post-translational modifications, into consideration^44–46^. In keeping with this, VHL-Homo-PROTAC has been shown to induce degradation of the VHL long form, which harbors a disordered region at its N-terminus, but not the VHL short form^47^. Thus, this supports our idea that the disordered region of targeted proteins is required for efficient degradation by PROTACs.

**Table 1:** Disordered region prediction of proteins targeted by PROTAC.

No.	Target	E3	Potency (DC_50_)	Disordered region (>40 a.a)	DOI
1	ABL1	CRBN,VHL	100 nM	O	10.1002/anie.201507634
2	AKT	VHL	100–300 nM	X	10.1002/psc.2858
3	ALK	CRBN	10 nM	O	10.1016/j.ejmech.2018.03.071
4	AR	VHL	5 nM	O	10.1038/s42003-018-0105-8
5	BCL6	CRBN	<1 μM	O	10.1021/acschembio.8b00698
6	BCR/ABL	CRBN	0.5–1 μM	O	10.1002/anie.201507634
7	BRD4	CRBN	<10 nM	O	10.1016/j.chembiol.2015.05.009
8	BRD9	CRBN	5–50 nM	O	10.1002/anie.201611281
9	BTK	CRBN	25 nM	O	10.1021/acs.biochem.8b00391
10	CDK4	CRBN	10–100 nM	X	10.1039/c9cc00163h
11	CDK6	CRBN	10–100 nM	X	10.1039/c9cc00163h
12	CDK8	CRBN	~100 nM	O	10.1021/acsmedchemlett.8b00011
13	c-MET	VHL	50 nM	X	10.1016/j.chembiol.2017.09.009
14	CRABP2	cIAP1	~1 μM	X	10.1016/j.febslet.2011.03.019
15	DHODH	VHL	0.1–1 μM	X	10.1002/chem.201702999
16	EGFR	VHL	22.3 nM	O	10.1016/j.chembiol.2017.09.009
17	ER	VHL	1 nM	O	10.1021/acs.jmedchem.8b01572
18	ERK2	CRBN	~1 μM	X	10.1021/acscentsci.6b00280
19	ERRa	VHL	100 nM	O	10.1038/nchembio.1858
20	FAK1	VHL	10 nM	O	10.1021/jacs.8b08008
21	FKBP12	CRBN	<100 nM	X	10.1126/science.aab1433
22	FLT3	VHL	2 nM	X	10.1021/jacs.8b10320
23	GCN5	CRBN	3 nM	O	10.1021/acschembio.8b00705
24	HDAC6	CRBN	100–300 nM	O	10.1016/j.bmcl.2018.05.057
25	HER2	VHL	25–100 nM	O	10.1016/j.chembiol.2017.09.009
26	PCAF	CRBN	1.5 nM	O	10.1021/acschembio.8b00705
27	RIPK2	VHL	1 nM	O	10.1038/nchembio.1858
28	Sirt2	CRBN	~0.5 μM	O	10.1021/acs.jmedchem.6b01872
29	TBK1	VHL	3 nM	X	10.1021/acs.jmedchem.7b00635
30	VHL	VHL	~10 nM	O	10.1038/s41467-017-00954-1

Based on a literature search, PROTACs with high potency (DC_50_ < 1 μM) were selected, and then analyzed them for a disordered region using the IUPred2A.

## Discussion

In this study, we developed a series of VHL-CRBN heterodimerizing PROTACs in which pomalidomide is linked with VHL ligand and demonstrated that these PROTACs exclusively induced the degradation of CRBN, but not VHL. Interestingly, no reduction in VHL level was observed with any of the VHL-CRBN heterodimerizing PROTACs that we tested. Why was only CRBN, and not VHL, degraded by these PROTACs? We first speculated that relative levels of the proteins might contribute to this biased protein degradation. To examine this, we treated cells overexpressing VHL, CRBN, or both with TD-158 and analyzed CRBN and VHL levels. In all cases, however, CRBN levels were reduced, whereas VHL levels remained similar or increased (Fig. 1E). Another possible explanation would be the number and accessibility of lysine residues for ubiquitination in each protein. The UPS requires a lysine residue on the target protein for ubiquitination. Human VHL protein possesses only three lysine residues, whereas human CRBN protein possesses 28 lysine residues. Therefore, it is likely that lysine residues on CRBN are ubiquitinated by the CRL2^VHL^ complex with a high probability once CRBN is present in close proximity to VHL. A three-dimensional structural analysis of the CRBN-VHL-PROTAC ternary complex (e.g., crystallization of the complex or Cryo-EM) will help to answer this question.

An intimate relationship between the disordered region of a protein and proteasome-mediated degradation has been demonstrated^25,28,29^. The unfolded or disordered region of a protein serves as an initiation site for processive unfolding and subsequent proteolysis by the proteasome^25,48,49^. Therefore, statistically, endogenous proteins containing an unfolded or disordered region of at least 40 amino acids in length are likely to be short lived^44–46^. In line with this, the half-life of full-length CRBN was shorter than that of D1 in cycloheximide chase experiments (Supplementary Fig. S6A,B). In the case of globular proteins, p97/VCP may help to expose unfolded regions for proteasomal degradation. However, CRBN degradation by VHL-CRBN heterodimerizing PROTACs was independent of the p97/VCP ATPase. Deletion of the disordered region of both CRBN and AR attenuated their degradation by PROTACs, and attachment of a disordered region to the globular CRBN deletion mutant or AR deletion mutant facilitated their degradation. However, in the case of VHL, attachment of a disordered region did not promote its degradation, indicating that the presence of a disordered region is not sufficient for degradation of all PROTAC-targeted proteins; there might be other elements in targeted proteins involved in determining degradability. In summary, the results obtained in this study explain the mechanism by which PROTACs degrade targeted proteins through the proteasome and provide a novel criterion for choosing degradable protein targets.

## Methods

### Cell culture, transfection, and establishment of stable cell lines

HEK293T (ATCC, CRL-11268) cells were cultured in Dulbecco’s Modified Eagle Medium (DMEM; Gibco, 11995) supplemented with 10% fetal bovine serum (FBS; Gibco, 16000) and an antimycotic (Gibco, 15240). Jurkat and 786-O cells were maintained in Roswell Park Memorial Institute (RPMI) 1640 medium (Gibco, 11875) supplemented with 10% FBS and an antimycotic. Cells were transfected using X-tremeGENE (Roche, 6366546001) according to the manufacturer’s protocol. Pre-designed siRNAs for silencing VHL were purchased from BIONEER. siRNA-mediated knockdown was performed using RNAiMAX (Invitrogen, 13778075) according to the manufacturer’s instructions. HEK293T cells were co-transfected with lentivirus VHL or shCUL2 plasmids together with pSPAX2 and pMD2.G using X-tremeGENE. After 48 h, media were collected, centrifuged, and filtered using a 0.45-μm syringe filter. After adding filtered media and 1 μM polybrene, the cells were incubated for 24 h. Infected cells were selected by incubating in the presence of puromycin for 7 to 10 d. Selected cells were confirmed by Western blot analysis.

### Plasmid constructs and site-directed mutagenesis

Xpress-CRBN full-length and deletion mutant constructs were generated by PCR from a pCNS-D2-CRBN plasmid purchased from 21 C Frontier Human Gene Bank. Mutations were introduced by site-directed mutagenesis using the QuikChange XL site-directed Mutagenesis Kit (Agilent, 200518–5) according to the manufacturer’s instructions. The unstructured region of CRBN (a.a. 1–80) was amplified by PCR and inserted into the *Bam*HI cleavage site upstream of the mutant constructs. The pEGFP-C1-AR construct (Addgene plasmid #28235) was subcloned into PCDNA4/His Max vector using a restriction enzyme-digested ΔN330 (a.a. 1–330 deletion) construct. The AR (a.a. 1–330) or CRBN (a.a. 1–80) was amplified by PCR and inserted into the *Pst*I or *Bam*HI restriction enzyme site upstream of the ΔN330 construct. Full-length CRBN and D1 deletion mutant were cloned into the PCDNA3.1 CHA vector, and the AR unstructured region (a.a.1–170) was inserted into the *Bam*HI restriction enzyme site upstream of the CRBN D1-HA construct.

### Antibodies and reagents

Antibodies against CRBN (HPA045910), Flag (F1804), and tubulin (T6074) were obtained from Sigma Aldrich. Antibodies against Aiolos (15103S), Ikaros (14859S), CHOP (2895S), and VHL (68547S) were obtained from Cell Signaling Technology. Antibodies against Cullin2 (ab166917), Cullin4a (ab72548), and DDB1 (ab109027) were obtained from Abcam. Antibodies against GAPDH (sc-47724) and HA (sc-805) were obtained from Santa Cruz Biotechnology. The Xpress (46-0528) antibody was obtained from Thermo Scientific. Flag M2 magnetic Beads (M8823, Sigma Aldrich) were used for immunoprecipitation. Bortezomib (504314) was obtained from Merck Millipore. EDTA-free Protease Inhibitor Cocktail (05056489001) was obtained from Roche.

### Protein purification and *In vitro* binding assay

Human full-length CRBN protein was cloned into pGEX 6P-1. For expression analysis, the plasmid was transformed into BL21-codon plus RIL competent cells and grown on Luria-Bertani (LB) agar plates. Human elongin B (a.a. 1–118) and elongin C (a.a. 17–112) genes were inserted into the pACYCDuet-1 plasmid. The VHL (a.a. 54–213) gene was cloned in the pGEX6P-1 vector. For expression analysis, pACYCDuet-1 and pGEX6P-1 vectors were co-transformed into *Escherichia coli* BL21 (DE3). Detailed methods are provided in supplementary material and methods sections. Purified CRBN, VHL/ELOB/ELOC and TD-158 were mixed in binding buffer (20 mM HEPES containing 100 mM NaCl, 5% [w/v] glycerol, 0.1% bovine serum albumin [BSA] and 0.1% Triton X-100), and incubated with glutathione magnetic agarose beads (78601, Thermo Scientific) at 4 °C for 6 h. The beads were washed with wash buffer A (20 mM HEPES containing 500 mM NaCl, 5% [w/v] glycerol, and 0.1% Triton X-100) and wash buffer B (20 mM HEPES containing 1 M NaCl, 5%[w/v] glycerol, and 0.1% Triton X-100) and then analyzed by immunoblotting and Coomassie Blue staining.

### LC–MS/MS analysis

Tandem mass tag (TMT)‐labeled peptides (1 μg) from each of the 24 fractions were dissolved in solvent A (2% acetonitrile and 0.1% formic acid); solvent B consisted of 98% acetonitrile and 0.1% formic acid. Nano‐LC‐MS/MS analyses were performed using a Q Exactive Mass Spectrometer (Thermo Scientific) equipped with an EASY‐Spray Ion Source and coupled to an EASY‐nLC 1000 (Thermo Scientific). Detailed methods are provided in supplementary material and methods sections.

### Statistical analysis

For mass spectrometry data analysis, FDRs of each protein for one sample t-test were then calculated using Storey method^50^. The DEPs were identified as the ones with FDR ≤0.05 and absolute log_2_-fold change ≥0.58 (1.5-fold). Cell viability data were analyzed using an independent Student’s t-test and considered significance at p < 0.01.

## Supplementary information

supplementary information

supplementary table S1
