# Supplementary material for: Disordered region of cereblon is required for efficient degradation by proteolysis-targeting chimera
Source: Sci Rep. 2019 Dec 23;9:19654. doi: 10.1038/s41598-019-56177-5 (PMC6928225; doi:10.1038/s41598-019-56177-5)

Disordered region of Cereblon is required for efficient degradation by Proteolysis Targeting Chimera

Kidae Kim[1,2], Dong Ho Lee[3], Sungryul Park[1,2], Seung-Hyun Jo[1,4], Bonsu Ku[1], Sung Goo Park[1,4], Byoung Chul Park[,1,2], Yeong Uk Jeon[3], Sunjoo Ahn[3,5], Chung Hyo Kang[6,7] Daehee Hwang[8], Sehyun Chae[9], Jae Du Ha[3], Sunhong Kim [1,10*], Jong Yeon Hwang [3,5*], and Jeong-Hoon Kim[1,4*]

[1] Disease Target Structure Research Center, Korea Research Institute of Bioscience and Biotechnology (KRIBB), Daejeon 34141, Republic of Korea

[2] Department of Proteome Structural biology, KRIBB School of Bioscience, Korea University of Science and Technology, Daejeon 34113, Republic of Korea

[3] Therapeutics & Biotechnology, Korea Research Institute of Chemical Technology, Daejeon 34114, Republic of Korea

[4] Department of Functional Genomics, KRIBB School of Bioscience, Korea University of Science and Technology, Daejeon 34113, Republic of Korea

[5] Department of Medicinal Chemistry and Pharmacology, Korea University of Science and Technology, Daejeon 34113, Republic of Korea

[6] Bio & Drug Discovery Division, Korea Research Institute of Chemical Technology, Daejeon 34114, Republic of Korea

[7] College of Pharmacy, Chungnam National University, Daejeon 34134, Republic of Korea

[8] Department of Biological Sciences, Seoul National University, Seoul 08826, Republic of Korea

[9] Korea Brain Bank, Korea Brain Research Institute, Daegu 41062, Republic of Korea

[10] Department of Bio-Molecular Science, KRIBB School of Bioscience, Korea University of Science and Technology, Daejeon 34113, Republic of Korea

*Corresponding authors. E-mail: sunhong@kribb.re.kr (S. Kim), jyhwang@krict.re.kr (J.H. Hwang), and jhoonkim@kribb.re.kr (J.-H. Kim)

Keywords: CRBN, VHL, disordered region, PROTAC

Supplementary Information

Supplementary Methods

Materials and Methods

All chemicals were obtained from commercial vendors (Sigma Aldrich, TCI, Alfa Aesar, etc) and used without further purification. Solvents were dried by using an aluminum oxide column. Thin-layer chromatography was performed on pre-coated silica gel 60 F254 plates (Merck, art. 5715). Purification was carried out by normal phase column chromatography (MPLC, Silica gel cartridge with 230-400 mesh). ^{1}H and ^{13}C NMR spectra were recorded with Bruker Avance 300 and 500, using $CDCl_3$ or other deuterated solvents as an internal standard. LC/MS was detected on Agilent Technology 6130 Quadrupole LC/MS with electrospray ionization.

Synthesis

General synthetic method for 3

A solution of 3-fluorothalidomide **1** (1 eq.), linker **2** (1.2 eq.), and DIPEA (1.2) in DMSO was stirred at 90 ^{o}C overnight,. The reaction mixture was diluted with water and extracted with EtOAc. The organic layer was washed with brine, dried over $MgSO_4$, and concentrated. The crude residue was purified on silica gel column chromatography using EtOAc/Hex to afford the desired product **3**.

***tert*-butyl 9-((2-(2,6-dioxopiperidin-3-yl)-1,3-dioxoisoindolin-4-yl)amino)nonanoate (3a)**

The title of compound was obtained in 59% as a green oil from *tert*-butyl 9-aminononaoate **2a** using the general synthetic procedure of **3**. ^{1}H NMR (300 MHz, $CDCl_3$) δ 8.12 (s, 1H), 7.49 (dd, J = 8.5, 7.1 Hz, 1H), 7.12–7.05 (m, 1H), 6.88 (d, J = 8.5 Hz, 1H), 6.23 (t, J = 5.6 Hz, 1H), 4.98–4.86 (m, 1H), 3.32–3.20 (m, 2H), 2.94–2.65 (m, 3H), 2.25–2.17 (m, 2H), 2.17–2.08 (m, 1H), 1.74–1.52 (m, 6H), 1.44 (s, 9H), 1.42–1.30 (m, 6H).

***tert*-butyl 11-((2-(2,6-dioxopiperidin-3-yl)-1,3-dioxoisoindolin-4-yl)amino)undecanoate (3b)**

The title of compound was obtained in 38% as a green solid from *tert*-butyl 11-aminoundecanoate

2b using the general synthetic procedure of **3**. ^{1}H NMR (300 MHz, $CDCl_3$) δ 8.03 (s, 1H), 7.49 (t, *J* = 7.9 Hz, 1H), 7.09 (d, *J* = 7.3 Hz, 1H), 6.92–6.86 (m, 1H), 6.23 (t, *J* = 5.1 Hz, 1H), 4.96–4.88 (m, 1H), 3.26 (q, *J* = 6.1 Hz, 2H), 2.96–2.69 (m, 3H), 2.20 (t, *J* = 7.5 Hz, 2H), 2.17–2.10 (m, 1H), 1.66 (p, *J* = 7.4 Hz, 2H), 1.44 (s, 9H), 1.43–1.38 (m, 2H), 1.37–1.24 (m, 12H).

***tert*-butyl 2-(2-(2-(2-((2-(2,6-dioxopiperidin-3-yl)-1,3-dioxoisoindolin-4-yl)amino)ethoxy)ethoxy)ethoxy)acetate (3c)**

The title of compound was obtained in 32% as a green oil from *tert*-butyl 2-(2-(2-(2-aminoethoxy)ethoxy)ethoxy)acetate **2c** using the general synthetic procedure of **3**. ^{1}H NMR (300 MHz, $CDCl_3$) δ 8.22 (s, 1H), 7.49 (dd, *J* = 8.5, 7.1 Hz, 1H), 7.10 (d, *J* = 7.1 Hz, 1H), 6.93 (d, *J* = 8.5 Hz, 1H), 6.50 (t, *J* = 5.6 Hz, 1H), 4.99–4.85 (m, 1H), 4.02 (s, 2H), 3.79–3.66 (m, 10H), 3.47 (q, *J* = 5.5 Hz, 2H), 2.95–2.65 (m, 3H), 2.19–2.08 (m, 1H), 1.47 (s, 9H).

General tert-butylester deprotection for 4

To a solution of **3** (100 mg) in DCM (1 mL) was added TFA (1 mL) and stirred at rt for 1 h. The reaction mixture was concentrated in vacuum to afford **4** as a crude product. The crude was used in the next step without purification after LC/MS analysis.

General synthetic method for 6

To a solution of **4** (1 eq.), VHL ligand **5** (1 eq.), HATU (1.5 eq.) in DMF was DIPEA (2 eq.) and stirred at rt overnight. The reaction mixture was diluted with water and extracted with EtOAc. The organic layer was washed with brine, dried over $MgSO_4$, and concentrated. The crude residue was purified on PREP-TLC using MeOH/DCM (5% as an eluent) to afford the desired product **6**.

(2S,4R)-1-((2S)-2-(9-((2-(2,6-dioxopiperidin-3-yl)-1,3-dioxoisoindolin-4-yl)amino)nonanamido)-3,3-dimethylbutanoyl)-4-hydroxy-N-(4-(4-methylthiazol-5-yl)benzyl)pyrrolidine-2-carboxamide (6a)

The title of compound was obtained in 35% as a yellow solid. ^{1}H NMR (300 MHz, $CDCl_3$) δ 10.63 (s, 0.5H), 10.37 (s, 0.5H), 10.32 (s, 0.5H), 10.29 (s, 0.5H), 8.67 (s, 1H), 7.68–7.53 (m, 1H), 7.44 (t, *J* = 7.8 Hz, 1H), 7.38–7.30 (m, 3H), 7.03 (d, *J* = 7.1 Hz, 1H), 6.94–6.76 (m, 2H), 6.22 (s, 1H), 4.98–4.78 (m, 1H), 4.69–4.44 (m, 4H), 4.34–4.09 (m, 2H), 4.05–3.90 (m, 1H), 3.69–3.56 (m, 1H), 3.28–

3.11 (m, 2H), 2.83–2.56 (m, 3H), 2.52–2.32 (m, 4H), 2.22–1.96 (m, 4H), 1.66–1.44 (m, 4H), 1.40–1.09 (m, 7H), 0.91 (s, 9H); ^{1}H NMR (300 MHz, Methanol-d_4) δ 8.88 (s, 1H), 7.60–7.35 (m, 5H), 7.10–6.91 (m, 2H), 5.13–5.01 (m, 1H), 4.71–4.48 (m, 4H), 4.44–4.29 (m, 1H), 3.99–3.87 (m, 1H), 3.87–3.76 (m, 1H), 3.31–3.27 (m, 1H), 2.98–2.66 (m, 3H), 2.48 (s, 3H), 2.39–2.19 (m, 3H), 2.19–2.00 (m, 2H), 1.74–1.51 (m, 4H), 1.51–1.21 (m, 10H), 1.04 (s, 9H); LC/MS (ESI) *m/z* $[M+H]^+$ 842.6, $[M-H]^-$ 840.7

(2S,4R)-1-((2S)-2-(11-((2-(2,6-dioxopiperidin-3-yl)-1,3-dioxoisoindolin-4-yl)amino)undecanamido)-3,3-dimethylbutanoyl)-4-hydroxy-N-(4-(4-methylthiazol-5-yl)benzyl)pyrrolidine-2-carboxamide (6b)

The title of compound was obtained in 55% as a yellow solid. ^{1}H NMR (500 MHz, $CDCl_3$) δ 9.97–9.86 (m, 1H), 8.68 (s, 1H), 7.53 (t, *J* = 6.2 Hz, 1H), 7.48 (t, *J* = 7.8 Hz, 1H), 7.39–7.32 (m, 4H), 7.10–7.04 (m, 1H), 6.88 (d, *J* = 8.5 Hz, 1H), 6.71–6.61 (m, 1H), 6.30–6.20 (m, 1H), 4.95–4.82 (m, 1H), 4.72–4.48 (m, 4H), 4.35–4.25 (m, 1H), 4.14–4.04 (m, 1H), 3.63 (d, *J* = 11.3 Hz, 1H), 3.52–3.38 (m, 1H), 3.34–3.20 (m, 2H), 2.88–2.63 (m, 3H), 2.57–2.46 (m, 4H), 2.23–2.05 (m, 4H), 1.70–1.59 (m, 2H), 1.60–1.47 (m, 2H), 1.47–1.36 (m, 2H), 1.36–1.29 (m, 2H), 1.29–1.18 (m, 8H), 0.97–0.85 (m, 9H); LC/MS (ESI) *m/z* 870.2 $[M+H]^+$, 868.1 $[M-H]^-$

(2S,4R)-1-((2S)-2-(tert-butyl)-14-((2-(2,6-dioxopiperidin-3-yl)-1,3-dioxoisoindolin-4-yl)amino)-4-oxo-6,9,12-trioxa-3-azatetradecanoyl)-4-hydroxy-N-(4-(4-methylthiazol-5-yl)benzyl)pyrrolidine-2-carboxamide (6c)

The title of compound was obtained in 21% as a yellow solid. ^{1}H NMR (300 MHz, $CDCl_3$) δ 9.81–9.72 (m, 1H), 8.70 (s, 1H), 7.57–7.46 (m, 2H), 7.41-7.31 (m, 5H), 7.11 (d, *J* = 7.1 Hz, 1H), 6.90 (d, *J* = 8.5 Hz, 1H), 6.54 (d, *J* = 4.1 Hz, 1H), 4.95–4.82 (m, 1H), 4.76–4.66 (m, 1H), 4.66–4.50 (m, 3H), 4.38–4.27 (m, 1H), 4.19–3.92 (m, 3H), 3.78–3.59 (m, 11H), 3.51–3.37 (m, 2H), 2.90–2.62 (m, 3H), 2.53 (d, *J* = 1.2 Hz, 3H), 2.52–2.41 (m, 1H), 2.22–2.09 (m, 1H), 1.91 (brs, merged with H_2O, 1H), 0.97 (s, 9H).

(2S,4R)-1-((2S)-2-(7-((2-(2,6-dioxopiperidin-3-yl)-1,3-dioxoisoindolin-4-yl)amino)heptanamido)-3,3-dimethylbutanoyl)-4-hydroxy-N-(4-(4-methylthiazol-5-yl)benzyl)pyrrolidine-2-carboxamide (6d)

^{1}H NMR ($CDCl_3$, 300MHz) δ 11.36 (s, 1H), 8.69 (s, 1H), 7.94 – 7.83 (m, 1H), 7.50 (dd, *J* = 8.5, 7.2 Hz, 1H), 7.41 – 7.30 (m, 4H), 7.12 (t, *J* = 8.3 Hz, 2H), 6.93 (d, *J* = 8.5 Hz, 1H), 6.41 – 6.33 (m, 1H), 4.92 – 4.85 (m, 1H), 4.73 – 4.55 (m, 4H), 4.26 (dd, *J* = 14.9, 4.8 Hz, 1H), 4.09 (d, *J* = 11.6 Hz, 1H), 3.66 (dd, *J* = 11.4, 3.5 Hz, 1H), 3.49 – 3.39 (m, 1H), 3.31 – 3.20 (m, 2H), 2.84 – 2.69 (m, 2H), 2.68 – 2.56 (m, 1H), 2.56 – 2.47 (m, 4H), 2.21 – 2.06 (m, 4H), 1.94 (s, 1H), 1.76 – 1.66 (m, 1H), 1.65 – 1.52 (m, 3H), 1.42 – 1.29 (m, 4H), 1.29 – 1.18 (m, 4H), 0.89 (s, 9H); LC/MS (ESI) *m/z* 814.6 $[M+H]^+$, 812.7 $[M-H]^-$

(2S,4S)-1-((2S)-2-(11-((2-(2,6-dioxopiperidin-3-yl)-1,3-dioxoisoindolin-4-yl)amino)undecanamido)-3,3-dimethylbutanoyl)-4-hydroxy-N-(4-(4-methylthiazol-5-yl)benzyl)pyrrolidine-2-carboxamide (8)

The title of compound was obtained in 34% as a yellow solid. ^{1}H NMR (500 MHz, Methanol-d_4) δ 9.04 (s, 1H), 7.93 (s, 2H), 7.56 (dd, *J* = 8.6, 7.1 Hz, 1H), 7.51–7.48 (m, 2H), 7.47–7.43 (m, 2H), 7.09–7.02 (m, 2H), 5.12–5.04 (m, 1H), 4.62–4.50 (m, 3H), 4.46–4.36 (m, 2H), 4.10–4.02 (m, 1H), 3.77–3.70 (m, 1H), 3.38–3.35 (m, 1H), 2.92–2.84 (m, 1H), 2.79–2.70 (m, 2H), 2.51 (s, 3H), 2.49–2.42 (m, 1H), 2.35–2.20 (m, 2H), 2.16–2.08 (m, 1H), 2.03–1.97 (m, 1H), 1.72–1.64 (m, 2H), 1.64–1.54 (m, 3H), 1.49–1.34 (m, 6H), 1.34–1.31 (m, 6H), 1.05 (s, 9H), LC/MS (ESI) *m/z* 870.31 $[M + H]^+$,

(2S,4R)-1-((2S)-2-(11-(2-((2-(2,6-dioxopiperidin-3-yl)-1,3-dioxoisoindolin-4-yl)amino)acetamido)undecanamido)-3,3-dimethylbutanoyl)-4-hydroxy-N-(4-(4-methylthiazol-5-yl)benzyl)pyrrolidine-2-carboxamide (6e)

The title of compound was obtained in 18% as a yellow solid. ^{1}H NMR (500 MHz, Chloroform-*d*) δ 8.68 (d, *J* = 6.2 Hz, 1H), 7.62–7.52 (m, 2H), 7.40–7.32 (m, 4H), 7.22 (t, *J* = 7.8 Hz, 1H), 6.84 (t, *J* = 7.8 Hz, 1H), 6.70–6.56 (m, 2H), 4.93–4.84 (m, 1H), 4.72–4.53 (m, 4H), 4.31–4.22 (m, 1H), 4.08 (d, *J* = 11.5 Hz, 1H), 4.05–3.87 (m, 2H), 3.70–3.62 (m, 1H), 2.86–2.60 (m, 3H), 2.50 (s, 3H), 2.50–2.44

(m, 1H), 2.22–2.01 (m, 4H), 1.60–1.48 (m, 2H), 1.49–1.38 (m, 2H), 1.34–1.23 (m, 3H), 1.23–0.97 (m, 13H), 0.93 (s, 9H). , LC/MS (ESI) *m/z* 927.2 $[M + H]^+$, 925.1 $[M - H]^-$

(2S,4R)-1-((2S)-2-(11-(2-((2-(2,6-dioxopiperidin-3-yl)-1,3-dioxoisoindolin-4-yl)oxy)acetamido)undecanamido)-3,3-dimethylbutanoyl)-4-hydroxy-N-(4-(4-methylthiazol-5-yl)benzyl)pyrrolidine-2-carboxamide (6f)

The title of compound was obtained in 24% as a white solid. ^1H NMR (500 MHz, Chloroform-*d*) δ 8.71–8.66 (m, 1H), 7.73 (q, *J* = 7.4 Hz, 1H), 7.63–7.60 (m, 0.5 H), 7.55–7.50 (m, 1.5H), 7.45 (t, *J* = 5.2 Hz, 1H), 7.38–7.30 (m, 4H), 7.23–7.17 (m, 0.5H), 4.97–4.90 (m, 0.5H), 4.87–4.80 (m, 1H), 4.70–4.51 (m, 6H), 4.32–4.18 (m, 1H), 4.12 (d, *J* = 11.6 Hz, 1H), 3.67–3.58 (m, 1H), 3.54–3.43 (m, 1H), 3.35–3.24 (m, 1H), 2.93–2.63 (m, 3H), 2.56–2.45 (m, 4H), 2.24–2.05 (m, 4H), 1.63–1.56 (m, 2H), 1.56–1.47 (m, 2H), 1.46–1.13 (m, 14H), 0.93 (s, 9H). , LC/MS (ESI) *m/z* 928.1 $[M + H]^+$, 926.1 $[M - H]^-$

N1-(2-(2,6-dioxopiperidin-3-yl)-1,3-dioxoisoindolin-4-yl)-N7-((S)-1-((2S,4R)-4-hydroxy-2-((4-(4-methylthiazol-5-yl)benzyl)carbamoyl)pyrrolidin-1-yl)-3,3-dimethyl-1-oxobutan-2-yl)heptanediamide (12)

^1H NMR (300 MHz, Chloroform-*d*) δ 9.90 (s, 1H), 9.46 (s, 1H), 8.76 (d, *J* = 8.4 Hz, 1H), 8.68 (s, 1 H), 7.72–7.67 (m, 1H), 7.55-7.50 (m, 2H), 7.36–7.33 (m, 4H), 6.64 (d, *J* = 8.7 Hz, 1H), 4.91–4.86 (m, 1H), 4.65–4.58 (m, 4H), 4.30–4.24 (m, 1H), 4.14–4.10 (m, 1H), 3.71-3.65 (m, 2H), 2.87–2.69 (m, 3H), 2.52 (s, 3H), 2.47 (t, *J* = 7.2 Hz, 2H), 2.30–2.21 (m, 2H), 2.17–2.12 (m, 2H), 1.82–1.73 (m, 2H), 1.70 (s, 3H), 1.67–1.60 (m, 2H), 0.94 (s, 9H). , LC/MS (ESI) *m/z* 828.7 $[M + H]^+$, 826.0 $[M - H]^-$

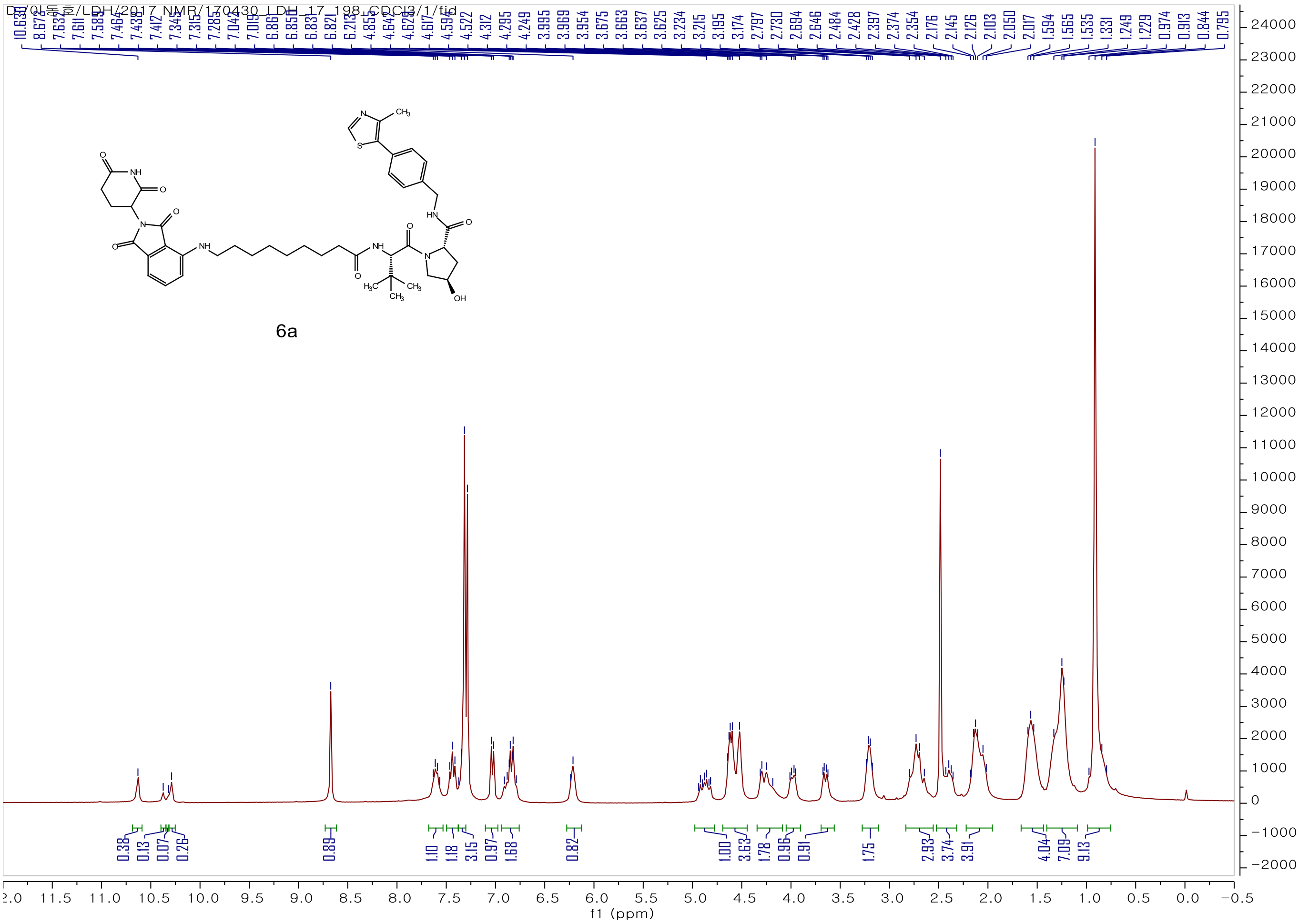

6a

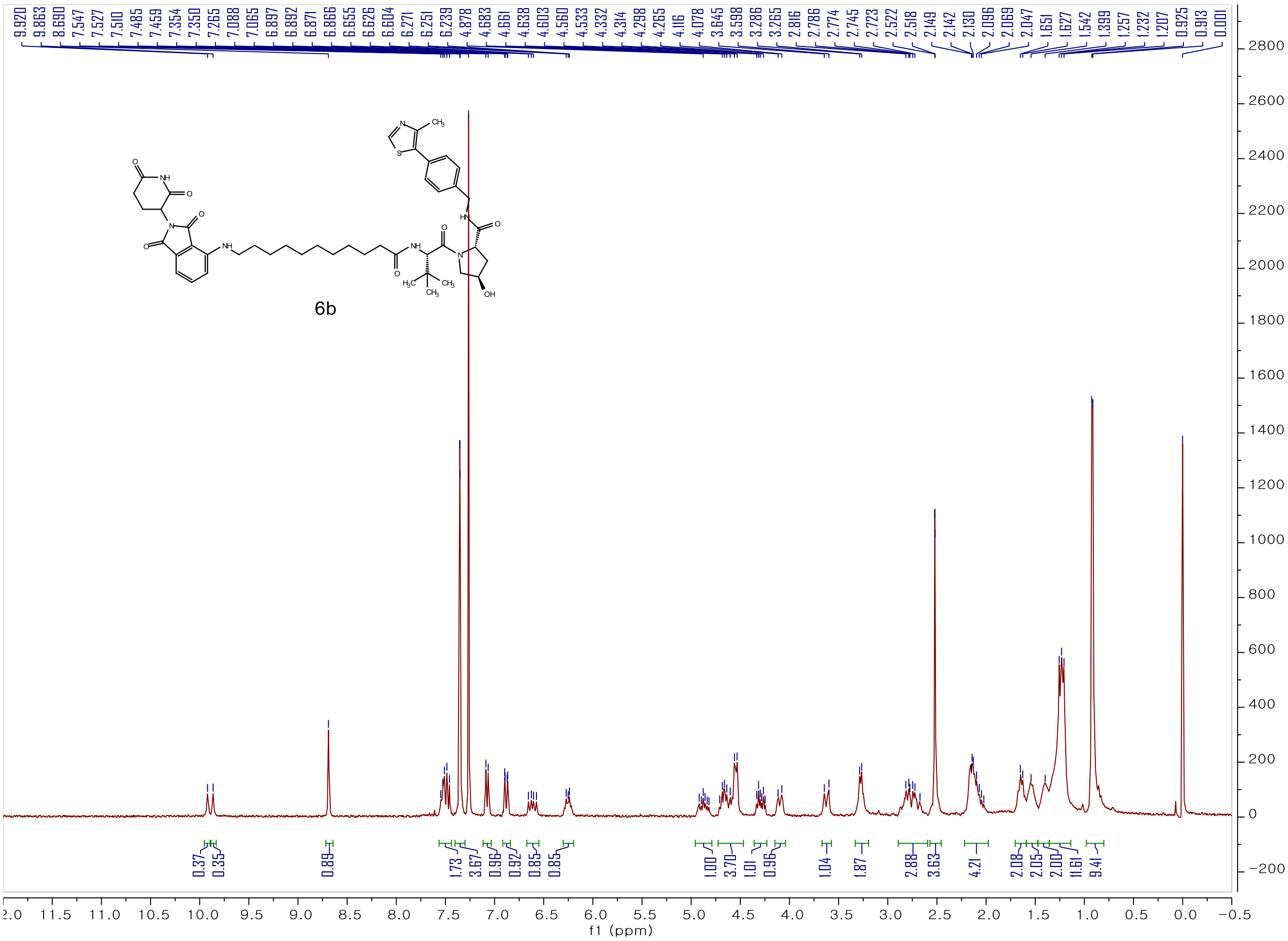

6b

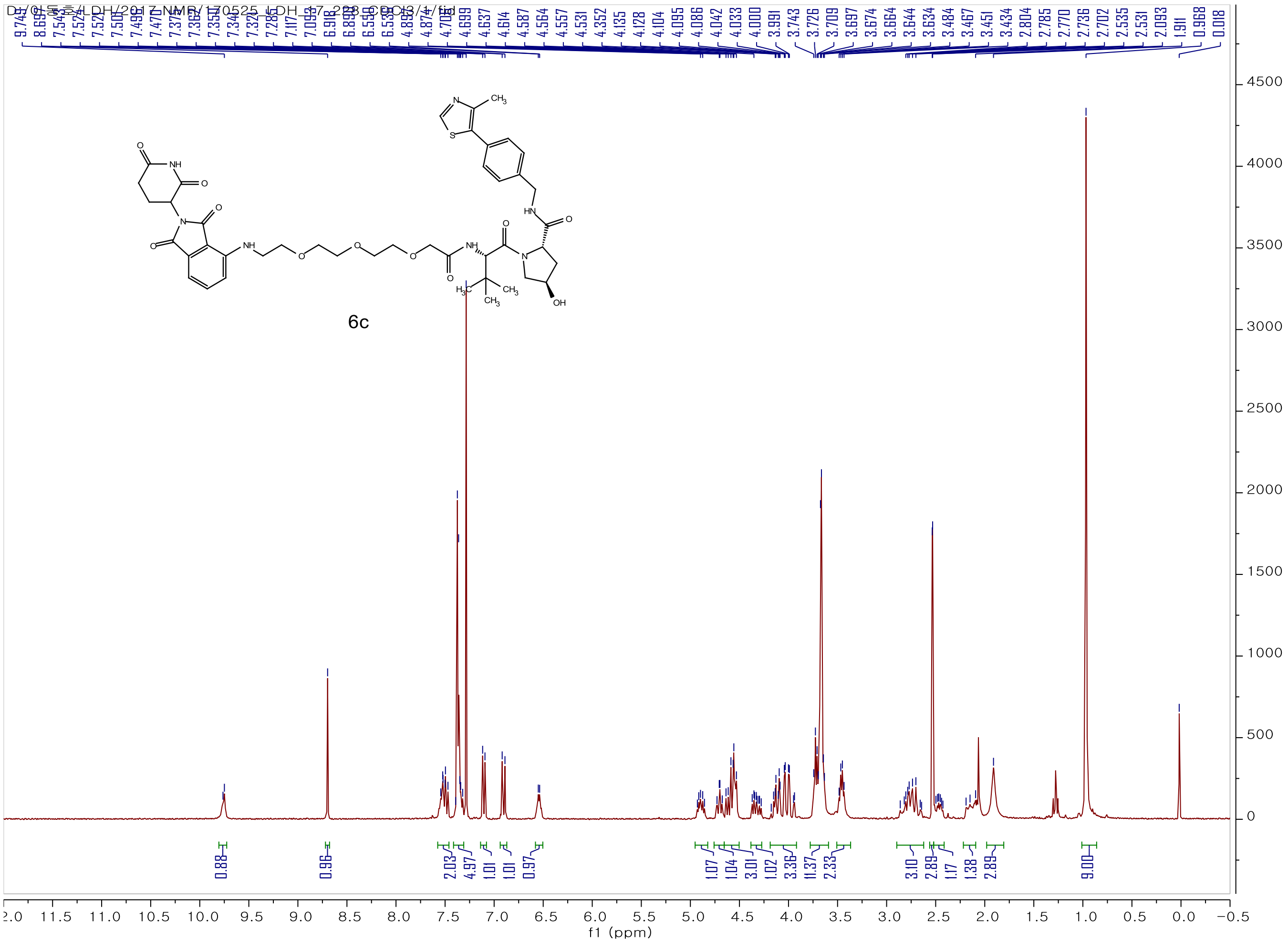
6c
f1 (ppm)

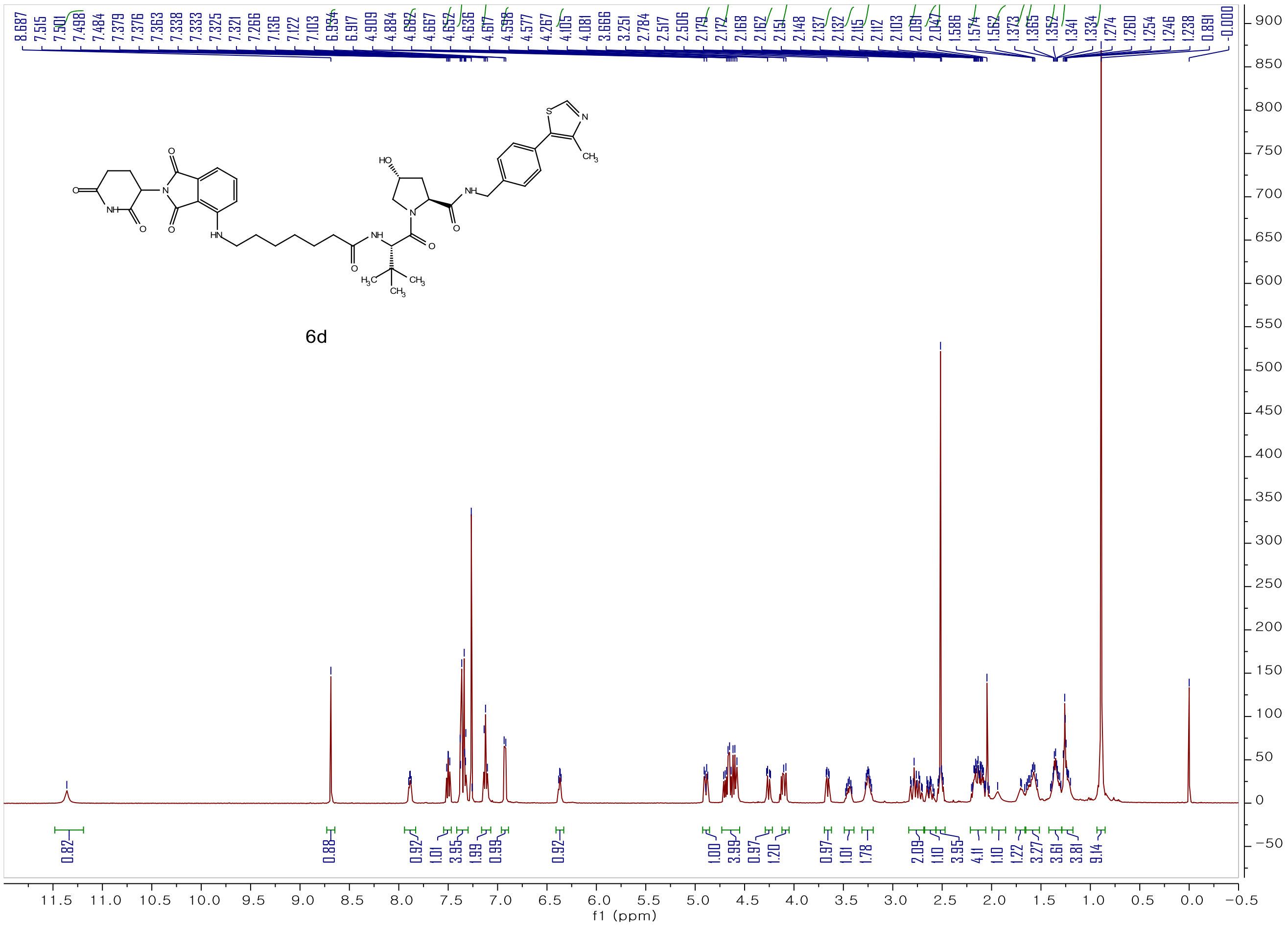
6d
f1 (ppm)

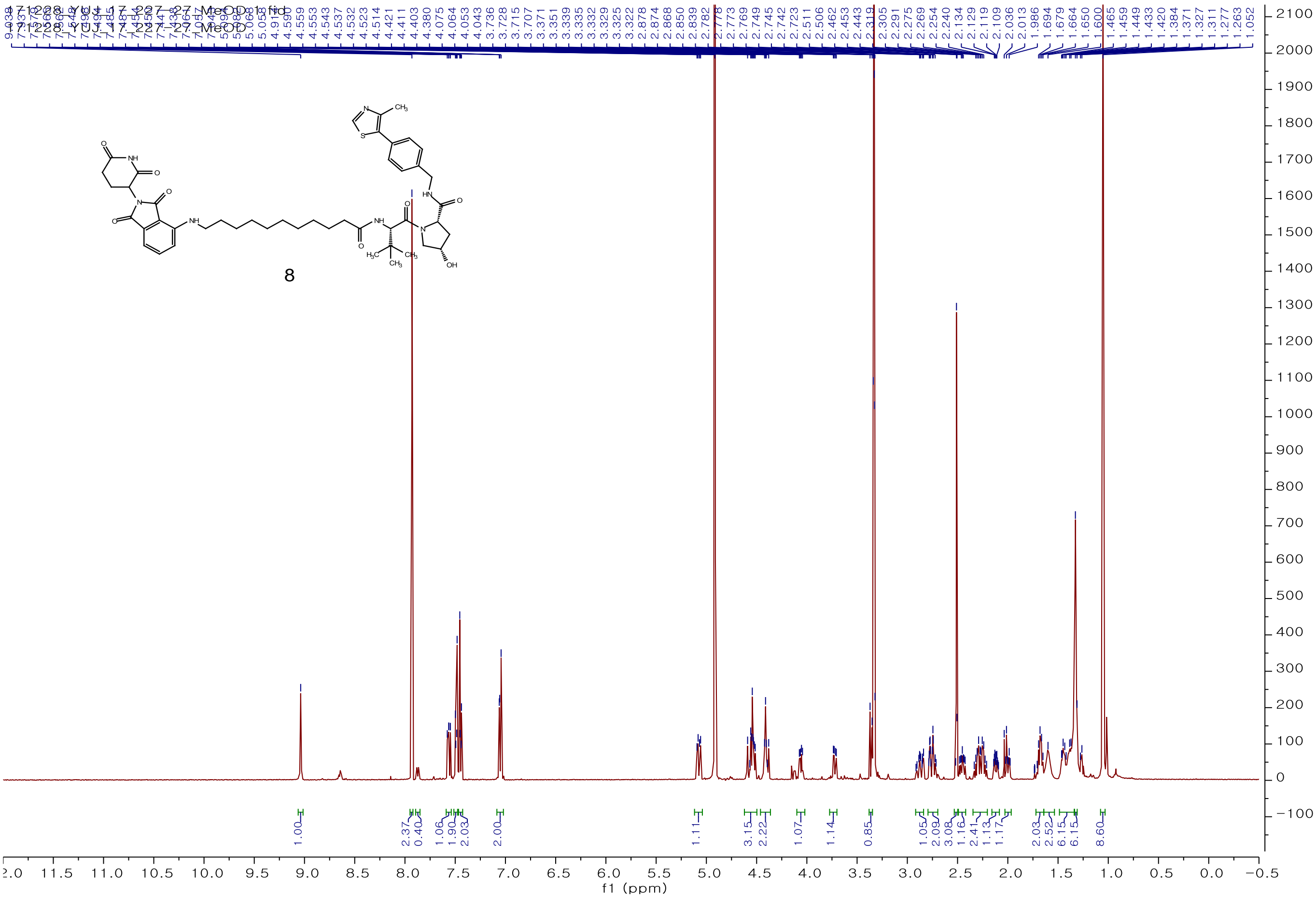
8
f1 (ppm)

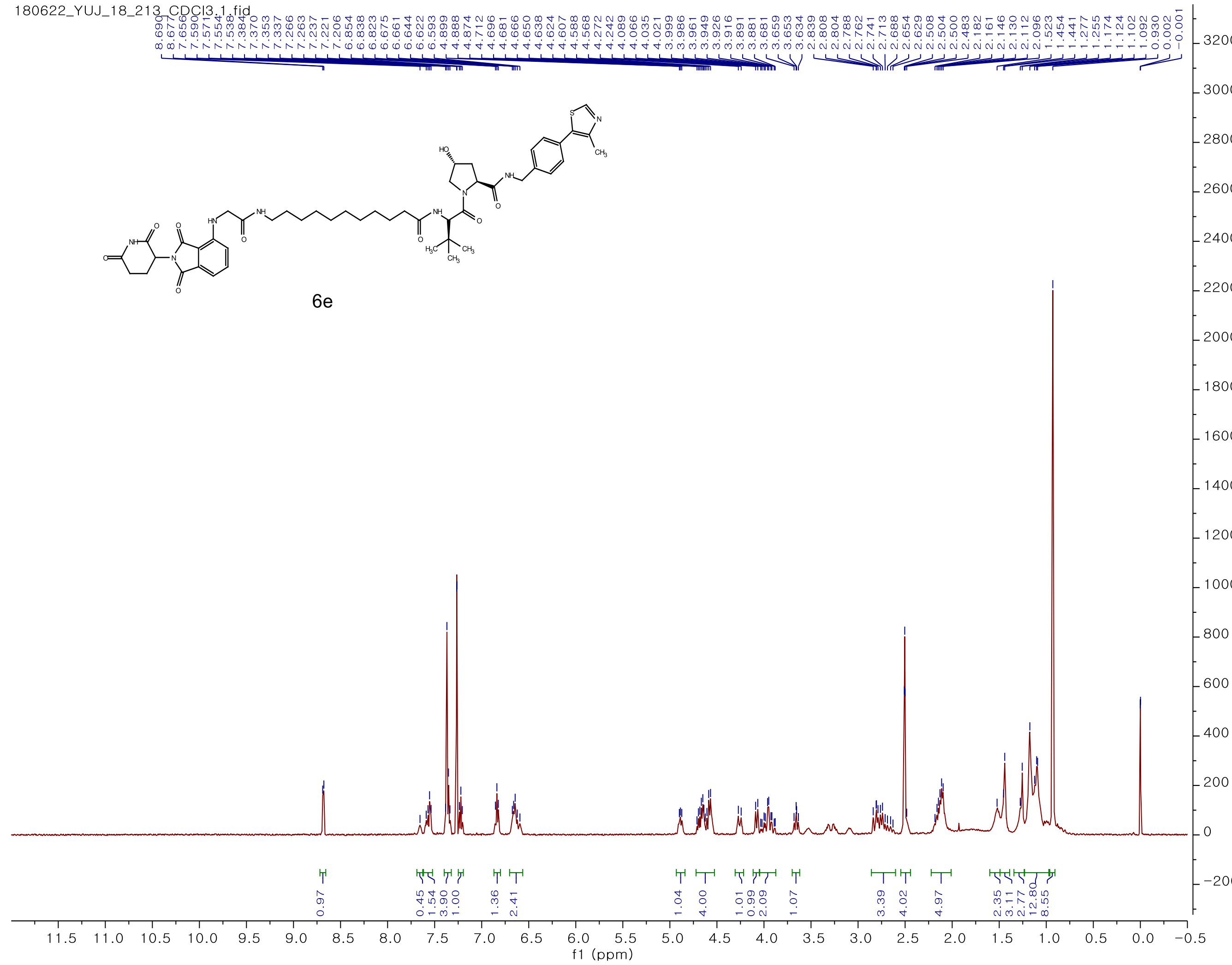
180622_YUJ_18_213_CDCl3.1.fid
6e
f1 (ppm)

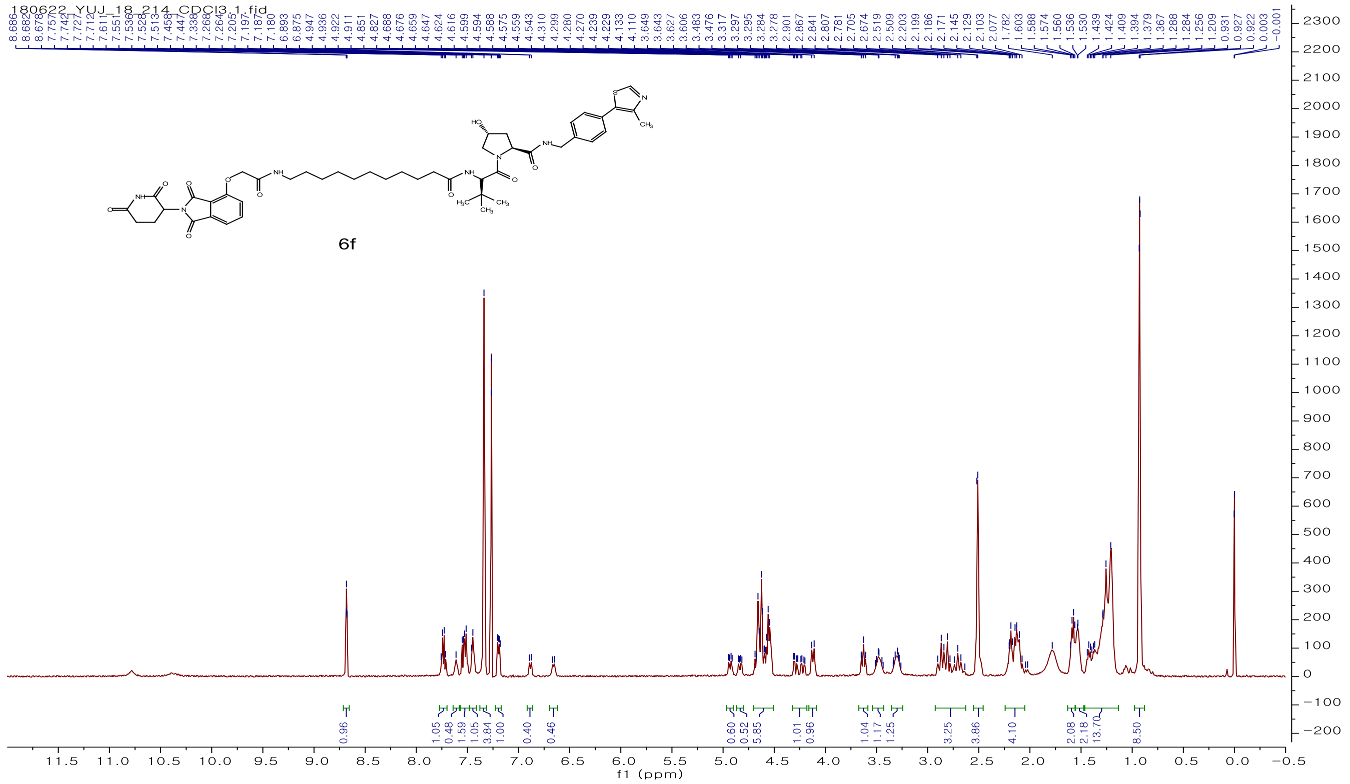

6f

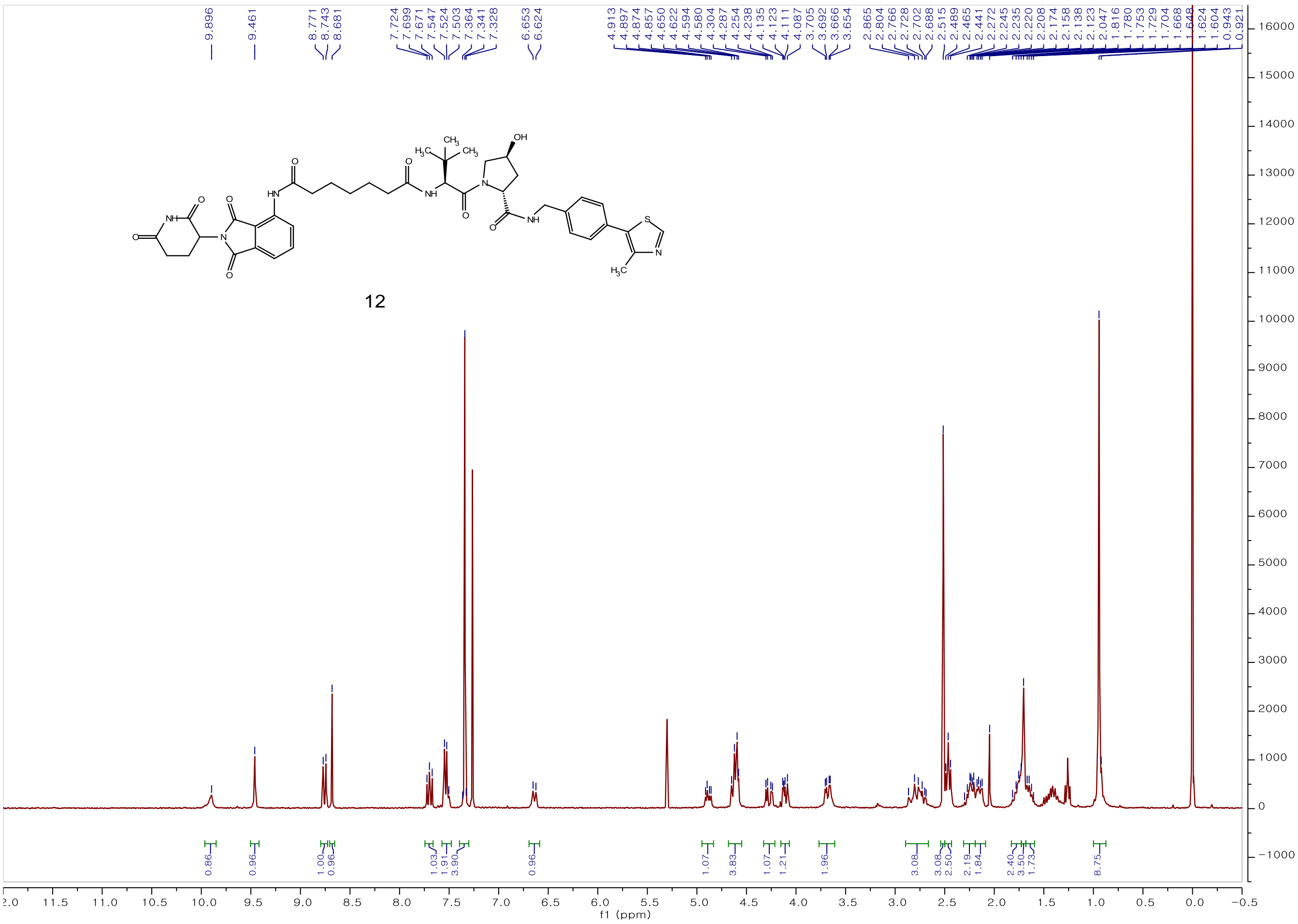

12

Bacterial protein expression and purification.

Human CRBN Full-length was cloned into pGEX 6P-1. For expression, plasmid was transformed to BL21-codon plus RIL competent cells and grown on LB agar plate. Single colony was picked for starter culture and grown in LB medium at 37°C shaking incubator for overnight. 1L of LB media was inoculated with 10ml starter culture and grown at 37°C shaking incubator until OD_{600} reaches 0.6. To induce protein expression 1 mM isopropyl β-d-1-thiogalactopyranoside (IPTG) was added to LB media and incubated at 18°C shaking incubator for overnight. Cells were harvested, washed with cold PBS, lysed with Extraction buffer (25 mM HEPES, 150 mM NaCl, 0.1% (v/v) igepal (NP40), 10% (w/v) glycerol, protease inhibitor cocktail, pH 8.0), sonicated, and centrifuged. The supernatant was incubated with GST agarose, washed, cleaved by PreScission protease (GE Healthcare, 27-0843-01). Human Elongin B (1–118 a.a) gene and human Elongin C (17–112 a.a) gene were inserted in the pACYCDuet-1 plasmid. VHL (54–213 a.a) gene was cloned in the pGEX6P-1 vector. For expression, pACYCDuet-1 and pGEX6P-1 vectors co-transformed in E. coli BL21 (DE3). E. coli were cultured in LB medium with 100 μg /m l of ampicillin and 25 μg /ml of chloramphenicol at 37 °C. When the cells grew and the value of O.D at 600 nm became 0.8, cells were induced by adding 0.6 μM isopropyl β-d-1-thiogalactopyranoside (IPTG) for 4 h, 37 °C. After induction, cells were collected by centrifugation, and pellets were sonicated in lysis buffer (50 mM NaH2PO4, 300 mM NaCl, 10 mM imidazole, pH 8.0). Lysates were ultra-centrifuged at 10,000 g, 4 °C for 1 h. Ni-NTA beads were mixed with lysate supernatant for 2 h. A mixture was loaded to polypropylene column and washed with wash buffer (50 mM NaH2PO4, 300 mM NaCl, 20 mM imidazole, pH 8.0). VHL/EloB/EloC (VBC) complex was eluted by elution buffer. To discard the high concentration of imidazole, eluted proteins were loaded to PD-10 column (GE Healthcare Life Sciences), and eluted by elution buffer (50 mM Tris-HCl, 200 mM NaCl, pH 7.4). The purified VBC complex was confirmed by SDS-PAGE and stored at −70 °C.

TMT Labeling and Peptide Fractionation

The proteins from three replicates were labeled with Amine-Reactive Tandem Mass Tag

Reagents (TMT6 Label Reagents, #90064; Thermo Scientific) according to the manufacturer's protocol. The peptide was solubilized in 100 mM TEAB. The TMT labels were reconstituted in 41 µL of acetonitrile prior to labeling, and 51 µL was added to the sample for labeling over 1 h at 25 °C. Eight microliters of 5% hydroxylamine were added to quench each reaction. After 15 min, the samples were combined. Finally, the labeled peptides were dried using vacuum centrifugation. To increase proteome coverage, Mid-pH RPLC was performed at a flow rate of 0.5 mL/min during 130 min gradient using solvent A (10 mM TEAB in water, pH 7.4) and solvent B (10 mM TEAB in 90 % ACN, pH 7.4). The gradient used is as follows: 0 % solvent B for 10 min, 0 - 5% solvent B in 10 min, 5 - 35 % in 60 min, 35 - 70 % in 15 min, 70 % for 10 min, 70 - 0 % in 10min, and finally held at 0% over 15 min. 96 fractions were collected in every minute from 15 min to 110 min and were non-contiguously concatenated into 24 fractions by pooling 4 fractions from each of early (#1-24), first mid (#25-48), second mid (#49-72), and late (#73-96) sections of fractions. The 24 fractions were dried in a vacuum centrifuge concentrator and stored at –80°C until LC-MS/MS experiments.

LC–MS/MS Analysis

TMT-labeled peptides (1 µg) from each of 24 fractions were dissolved in solvent A (solvent A consisted of 2% acetonitrile and 0.1% formic acid; solvent B consisted of 98% acetonitrile and 0.1% formic acid). Nano-LC-MS/MS analyses were performed using a Q Exactive Mass Spectrometer (Thermo Scientific) equipped with an EASY-Spray Ion Source and coupled to an EASY-nLC 1000 (Thermo Scientific). Peptides were loaded onto an Acclaim PepMap 100 pre-column (75 µm × 2 cm, C18, 3-µm particles, 100 Å pore size) and separated on an ES800 Easy-Spray column (50 cm × 75 µm inner diameter, PepMap C18, 3-µm particles, 100 Å pore size). A 180-min gradient was used at a flow rate of 300 nL min−1: from 2 to 40% solvent B over 120 min, from 40 to 80% solvent B over 30 min, 80% solvent B for 15 min, and 2% solvent B for 15 min. The temperature of the column was maintained at 35 °C. The electrospray voltage was 2.1 kV and the mass spectrometer was operated in data-dependent MS/MS mode. MS precursor scans (m/z range of 450–2000 Th) were acquired at an automated gain control (AGC) target value of 3.0×10^6, resolution of 70,000, and maximum

ion injection time of 240 ms. The MS/MS data for up to the ten most abundant ions were acquired in a data-dependent mode using higher energy collisional dissociation at a normalized collision energy of 27 with a fixed first mass of 120 Th at a resolution of 17,500, an AGC target value of 1.0 × 10^5, and a maximum injection time of 50 ms.

LC–MS/MS Data Analysis

For each MS/MS dataset, postexperiment monoisotopic mass refinement (PE-MMR) was used to process the MS/MS data, which was previously demonstrated to accurately assign precursor mass to the tandem mass spectrometric data [1]. The resulting MS/MS data from the PE-MMR process (i.e., mgf files) were subjected to a database search using the MS-GF+ search engine (v2017.01.13) [2] against the UniProt-Prot-Human-reference database (released November, 2017; 21,200 entries). Search parameters were set to precursor mass tolerance of 10 ppm, semitryptic, static modifications of carbamidomethylation (+57.0214 Da) to cysteine, and variable modification of TMT (+229.162932 Da) to N-termini and lysine and oxidation (+15.994915 Da) to methionine. The search results from the 24 MS/MS datasets were combined. The peptide spectrum matches (PSMs) were obtained using the false discovery rate (FDR) of 1%.

Identification of Differentially Expressed Proteins

The reporter ion intensities of the identified peptides were normalized using quantile-normalization methods [3]. We then selected the sets of proteins that have more than three non-redundant unique peptides. For the selected proteins, the relative protein abundances were computed from unique peptide intensities as previously described [4]. To identify the DEPs, we then performed one sample t-test using $\log_2$-fold changes in all peptides of each protein to compute the significance of T values. An empirical distribution of the null hypothesis (i.e., a protein is not differentially expressed) was estimated by performing all possible random permutations of the samples and then by applying the Gaussian kernel density estimation method to T values resulted from the random permutations. FDRs of each protein for one sample t-test were then calculated

using Storey method [5]. The DEPs were identified as the ones with FDR ≤ 0.05 and absolute log_2-fold change ≥ 0.58 (1.5-fold).

Pharmacokinetic studies in mice

In vivo PK of compounds was examined in male ICR mice (6-7 weeks, OrientBio, Kyunggi-do, South Korea). For TD-165, three doses (2, 10, and 50 mg/kg) were administered via intraperitoneal route. Dosing vehicles were composed of 47% PEG300, 3% Cremophor EL and 50% Saline. After the retro-orbital blood sample collection at each time point, protein precipitation was conducted for the plasma sample preparation by adding acetonitrile at a 9:1 ratio to the plasma. After vortexing (10 min) and centrifugation (13,000 rpm, 10 min, 4°C), the supernatant was transferred for LC/MS/MS analysis. The concentrations of test compounds were determined by LC/MS/MS on an Agilent 1200 HPLC system coupled to an Agilent 6460 triple quadrupole mass spectrometer equipped with ESI source (Agilent, Santa Clara, CA, USA) using imipramine as an internal standard (IS). The peak areas for all components were automatically integrated using Agilent 6460 Quantitative Analysis processing software. Pharmacokinetic and statistical analyses of plasma concentrations and statistical analysis of pharmacokinetic parameters were performed using non-compartmental analysis with Phoenix WinNonlin (v6.4; Pharsight Corp., Mountain View, CA, USA). The area under the plasma concentration-time curve from time 0 to infinity (AUCinf) was calculated by the trapezoidal rule with extrapolation to time infinity. The terminal T1/2 was calculated as ln2/λz, where λz was the first-order rate constant associated with the terminal (log-linear) portion of the curve. The Cmax and the time when it occurred (Tmax) were obtained by visual inspection of the plasma concentration-time curve.

Equilibrium solubility

Excess amount of solid compound were added to the deionized water and samples were incubated at 25'C for 24 h on an orbital shaker. After filtration using the syringe filter (0.2 μm pore size, Whatman plc, UK), the compound concentration in the filtrate was determined using the LC-

MSMS system.

Parallel artificial membrane permeability assay

Permeability of TD-165 was evaluated using the PAMPA Explorer Test SystemTM (Pion Inc.). PAMPA sandwich was formed from a 96-well filter plates coated with GIT-0 lipid solution (Pion Inc.). The initial donor sample concentration was 10 uM and the acceptor compartment was filled with acceptor sink buffer (Pion Inc.). Then the PAMPA sandwich was incubated for 4 h in the Gut-BoxTM. After the incubation, the compound concentration in acceptor and donor was determined by UV spectrophotometry with an Epoch plate reader instrument (Biotek Inc., Winnoski, VTS) and permeability coefficient was determined using the PAMPA Explorer software.

Plasma protein binding

The unbound fractions to the human and mouse plasma protein were estimated using a rapid equilibrium dialysis (RED) system following the manufacturer's protocol (Thermo Scientific, Waltham, MA). Briefly, plasma sample with TD-165 (100 μM) and PBS were placed into the assembled RED system and then, incubated at 37'C on an orbital shaker for 4 h. After the incubation, samples were obtained from the device, equal volume of blank plasma or PBS was added to avoid matrix effect. Protein precipitation was conducted for the ample preparation by adding acetonitrile and after centrifugation the drug concentration of supernatant were analyzed using the LC-MSMS system. The unbound fraction of the drug was calculated.

References

1 Shin, B. *et al.* Postexperiment monoisotopic mass filtering and refinement (PE-MMR) of tandem mass spectrometric data increases accuracy of peptide identification in LC/MS/MS. *Mol Cell Proteomics* **7**, 1124-1134, doi:10.1074/mcp.M700419-MCP200 (2008).

2 Kim, S. & Pevzner, P. A. MS-GF+ makes progress towards a universal database search tool for proteomics. *Nat Commun* **5**, 5277, doi:10.1038/ncomms6277 (2014).

3 Bolstad, B. M., Irizarry, R. A., Astrand, M. & Speed, T. P. A comparison of normalization methods for high density oligonucleotide array data based on variance and bias. *Bioinformatics* **19**, 185-193, doi:10.1093/bioinformatics/19.2.185 (2003).

4 Cox, J. & Mann, M. MaxQuant enables high peptide identification rates, individualized p.p.b.-range mass accuracies and proteome-wide protein quantification. *Nat Biotechnol* **26**, 1367-1372, doi:10.1038/nbt.1511 (2008).

5 Storey, J. D. & Tibshirani, R. Statistical significance for genomewide studies. *Proceedings of the National Academy of Sciences of the United States of America* **100**, 9440-9445, doi:10.1073/pnas.1530509100 (2003).

Supplementary Table S2. Pharmacokinetic parameters of TD-165 in male ICR mice (Mean ± SD, n=3)

Parameters	I.P., 2 mg/kg	I.P., 10 mg/kg	I.P., 50 mg/kg
T_{max} (h)	0.36 ± 0.24	0.70 ± 0.29	0.50 ± 0.00
C_{max} (μg/mL)	0.32 ± 0.09	1.80 ± 0.79	6.36 ± 1.86
$T_{1/2}$ (h)	3.71 ± 2.05	5.24 ± 1.82	2.78 ± 0.04
AUC_t (μg·h/mL)	0.53 ± 0.13	3.29 ± 0.39	13.90 ± 0.91
AUC_∞ (μg·h/mL)	0.57 ± 0.15	3.32 ± 0.40	14.00 ± 0.91

Supplementary Table S3. Physicochemical properties and plasma protein binding of TD-165

		TD-165	
Solubility		0.111	mg/mL
PAMPA Permeability		2.19×10^{-6}	cm/sec
Plasma protein binding	Mouse	99.9	%
	Human	99.8	%

Supplementary Figure S1.

A

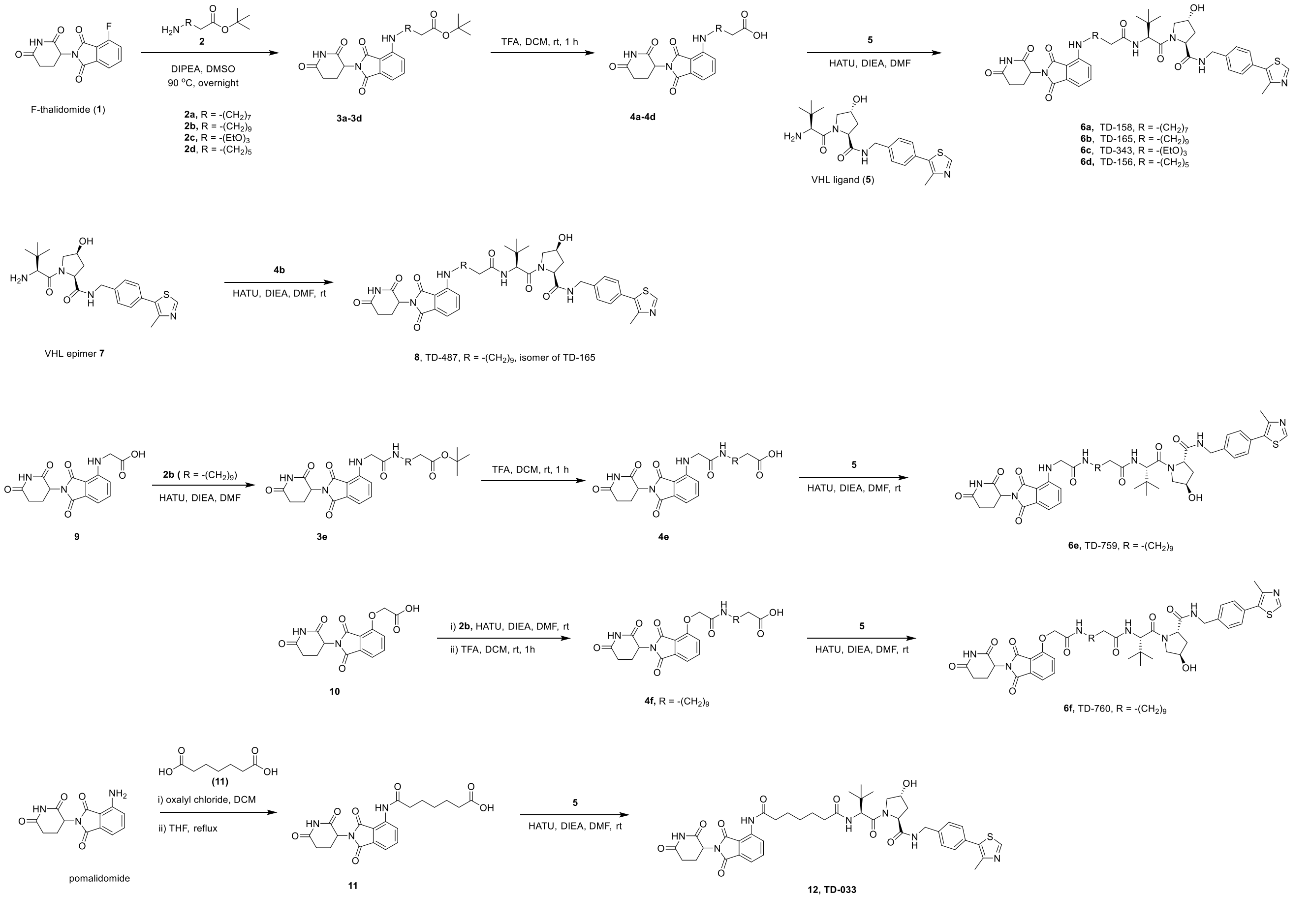

B

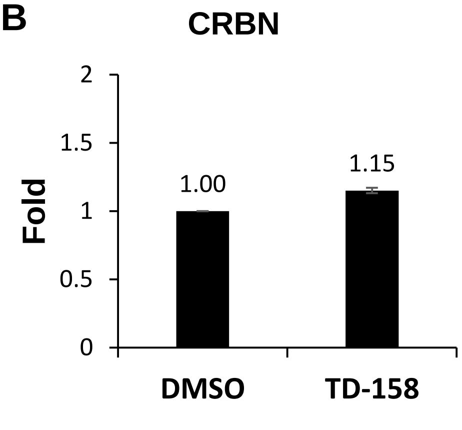

C

No.	Structure	DC_{50} (nM)	D_{max} (%)
**6a** (TD-158)	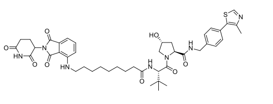	44.5	97.1
**6b** (TD-165)	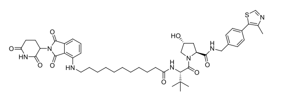	20.4	99.6
**6c** (TD-343)	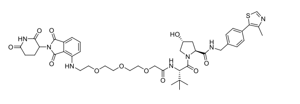	367.8	85.1
**6d** (TD-156)	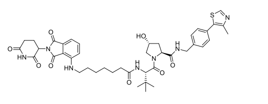	100.6	96.9
**6e** (TD-759)	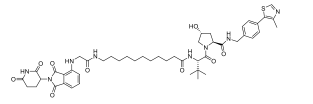	28.8	98.4
**6f** (TD-760)	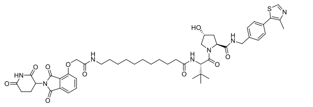	367.7	82.0
**8** (TD-487)	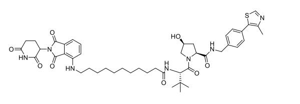	N.D	N.D
**12** (TD-033)	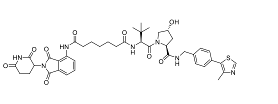	2546.1	77.9

Supplementary Figure S1.

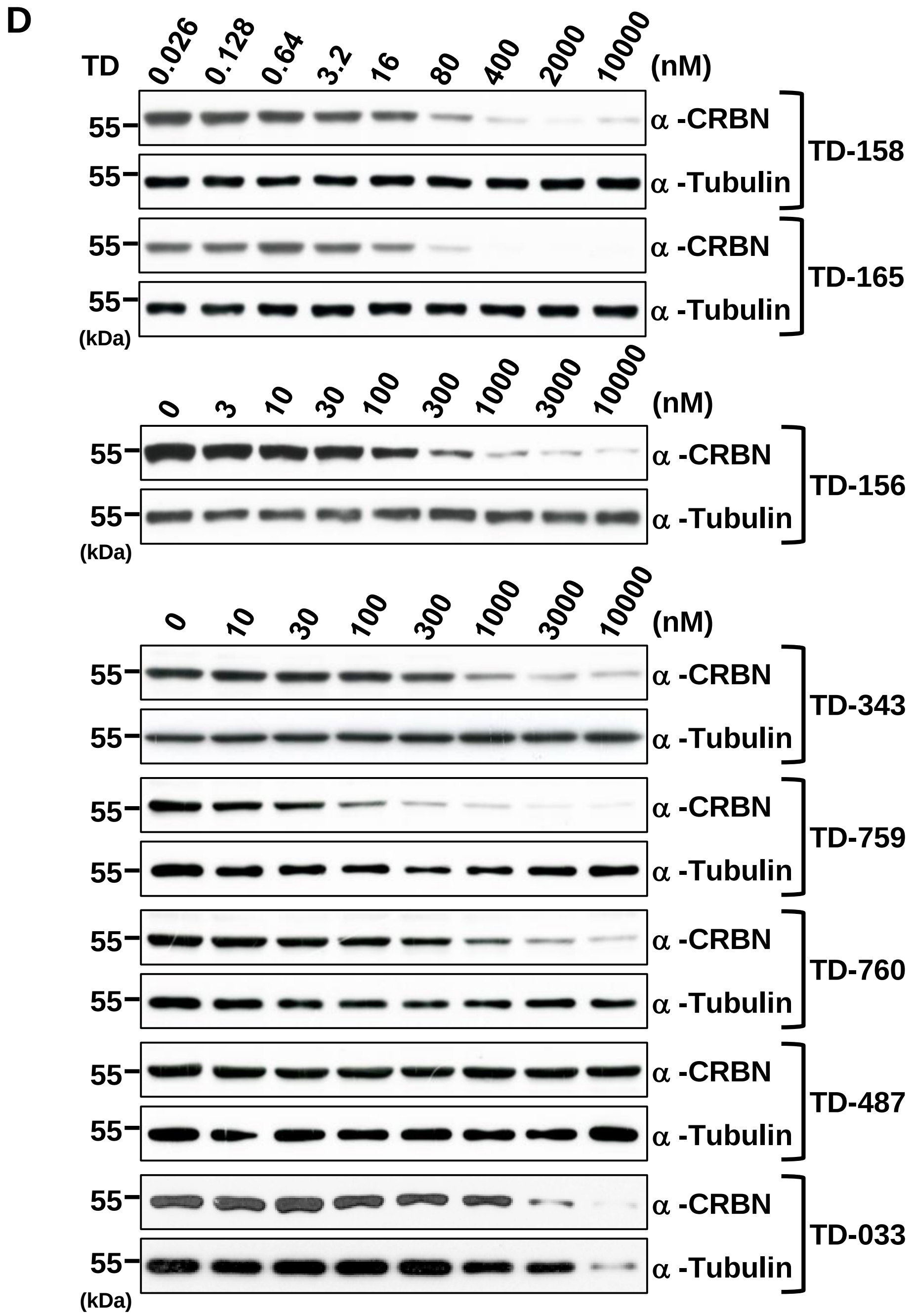

Supplementary Figure S1.

(A) Synthesis of the CRBN-PROTACs (B) HEK293T cells were treated with 500 nM TD-158 for 24 h. Total RNA was prepared from cells and analyzed by real time qPCR. (C) DC_{50}, D_{max} and structure of TD compounds. (D) HEK293T cells were treated with increasing concentrations of TD-158, TD-165, TD-156, TD-343, TD-759, TD-760, TD-487 or TD-033 for 24 h. CRBN protein levels were analyzed by immunoblotting.

Supplementary Figure S2.

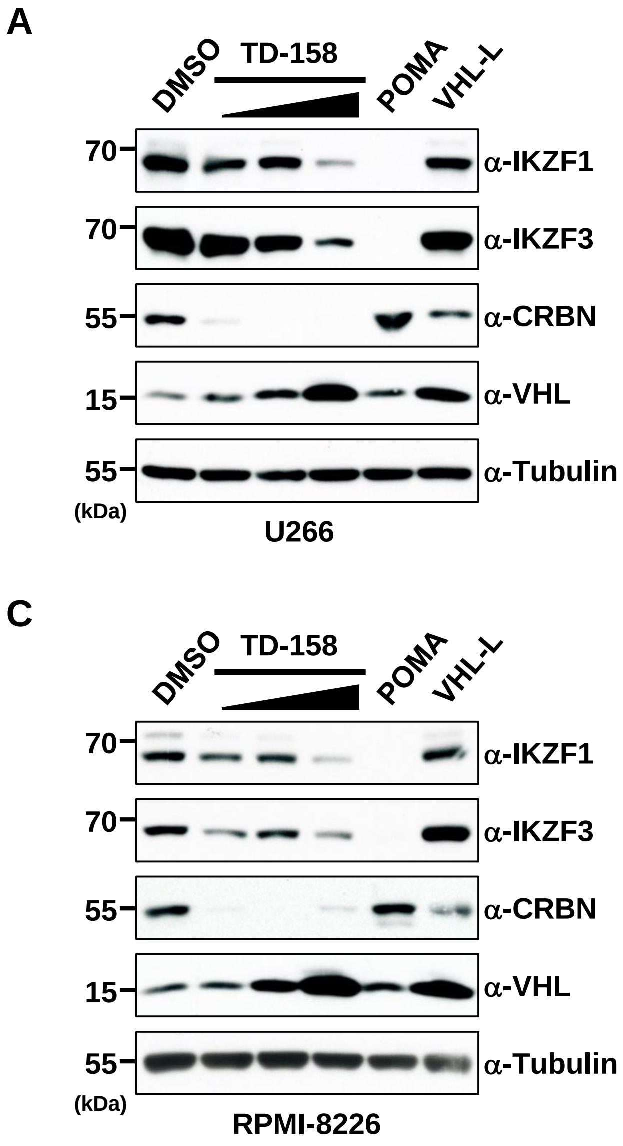

Supplementary Figure S2.

(A, B, C, D) U266 (A), IM-9 (B), RPMI-8226 (C), and NC37 (E) cells were treated with increasing concentrations of TD-158, Pomalidomide (1 μM) or VHL ligand (1 μM) for 48 h. Whole-cell lysates were analyzed by immunoblotting for the indicated proteins.

Supplementary Figure S3.

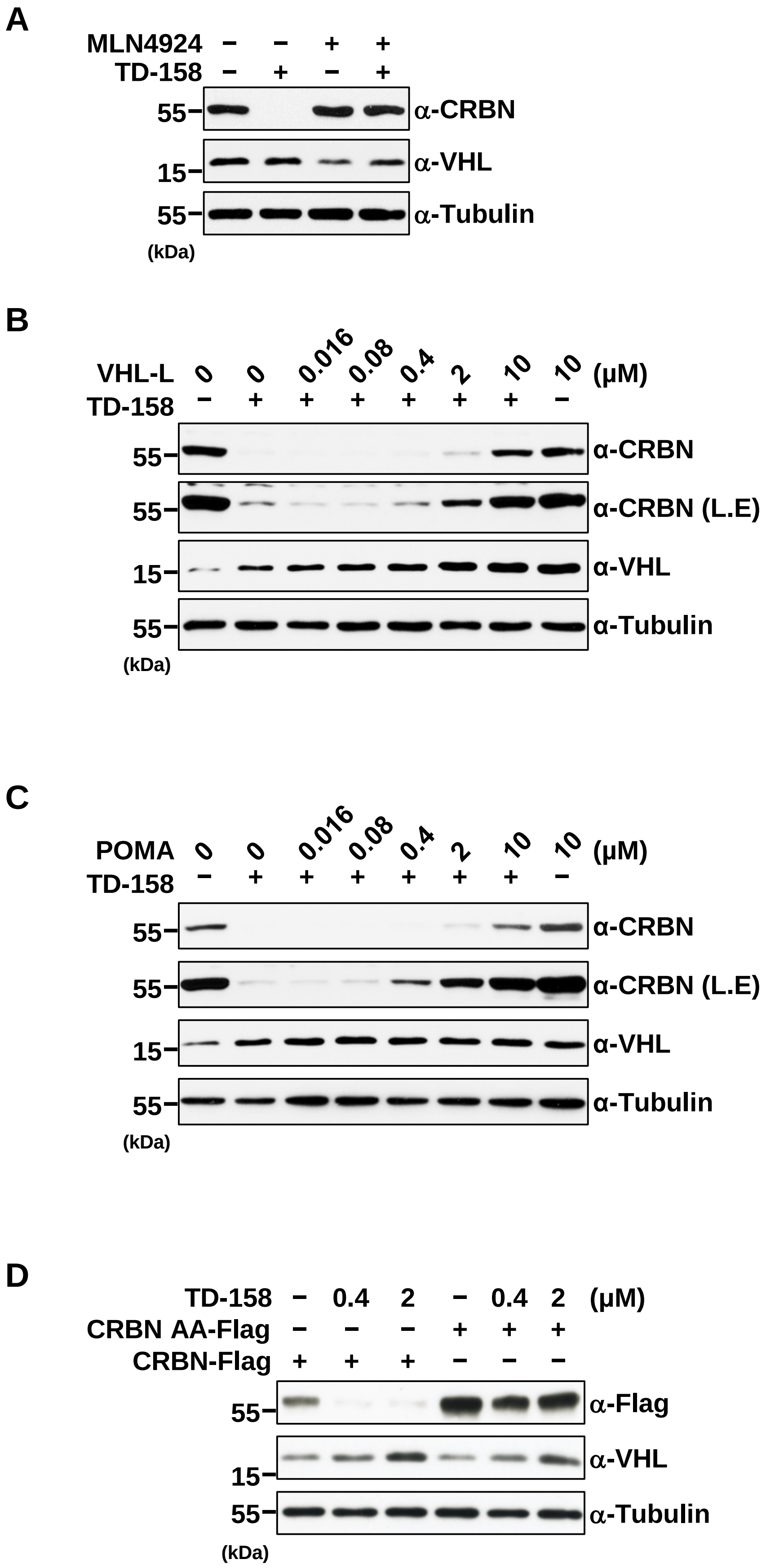

Supplementary Figure S3.

(A) HEK293T cells were treated with TD-158 (500 nM) for 16 h, followed by addition of MLN4924 (1 μM) or DMSO for 8 h Whole-cell lysates were analyzed by immunoblotting for the indicated proteins. (B, C) HEK293T cells were treated with TD-158 (500 nM) and increasing concentrations of VHL ligand (B) or Pomalidomide (C) for 24 h Whole-cell lysates were analyzed by immunoblotting for the indicated proteins. (D) Flag tagged CRBN Wild-type (CRBN-Flag) and CRBN Y384/W386A (CRBN AA-Flag) were expressed in the HEK293T cells. After 6 h, the cells were treated with DMSO or TD-158 (400 nM and 2 μM) for 48 h. Whole-cell lysates were analyzed by immunoblotting for the indicated proteins.

Supplementary Figure S4.

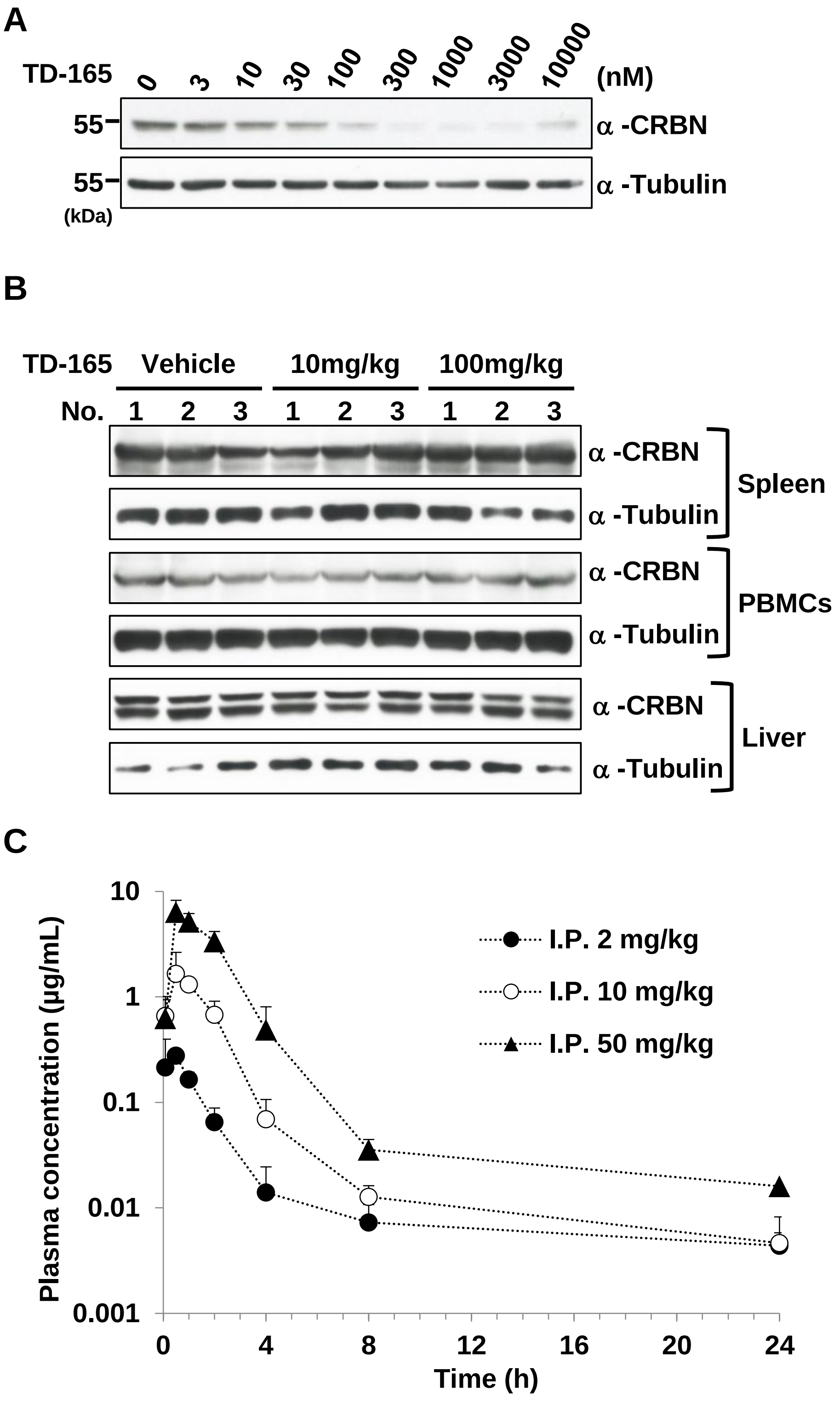

Supplementary Figure S4.

(A) MEF cells were treated with increasing concentrations of TD-165 for 48 h. CRBN protein levels were analyzed by immunoblotting. (B) Vehicle, 10 mg/kg or 100mg/kg of TD-165 were administered via intraperitoneal route. Spleen and Liver homogenates and peripheral blood mononuclear cell lysates were analyzed by immunoblotting. (C) Three doses (2, 10, and 50 mg/kg) of TD-165 were administered via intraperitoneal route and concentration of TD-165 was determined by LC/MS/MS.

Supplementary Figure S5.

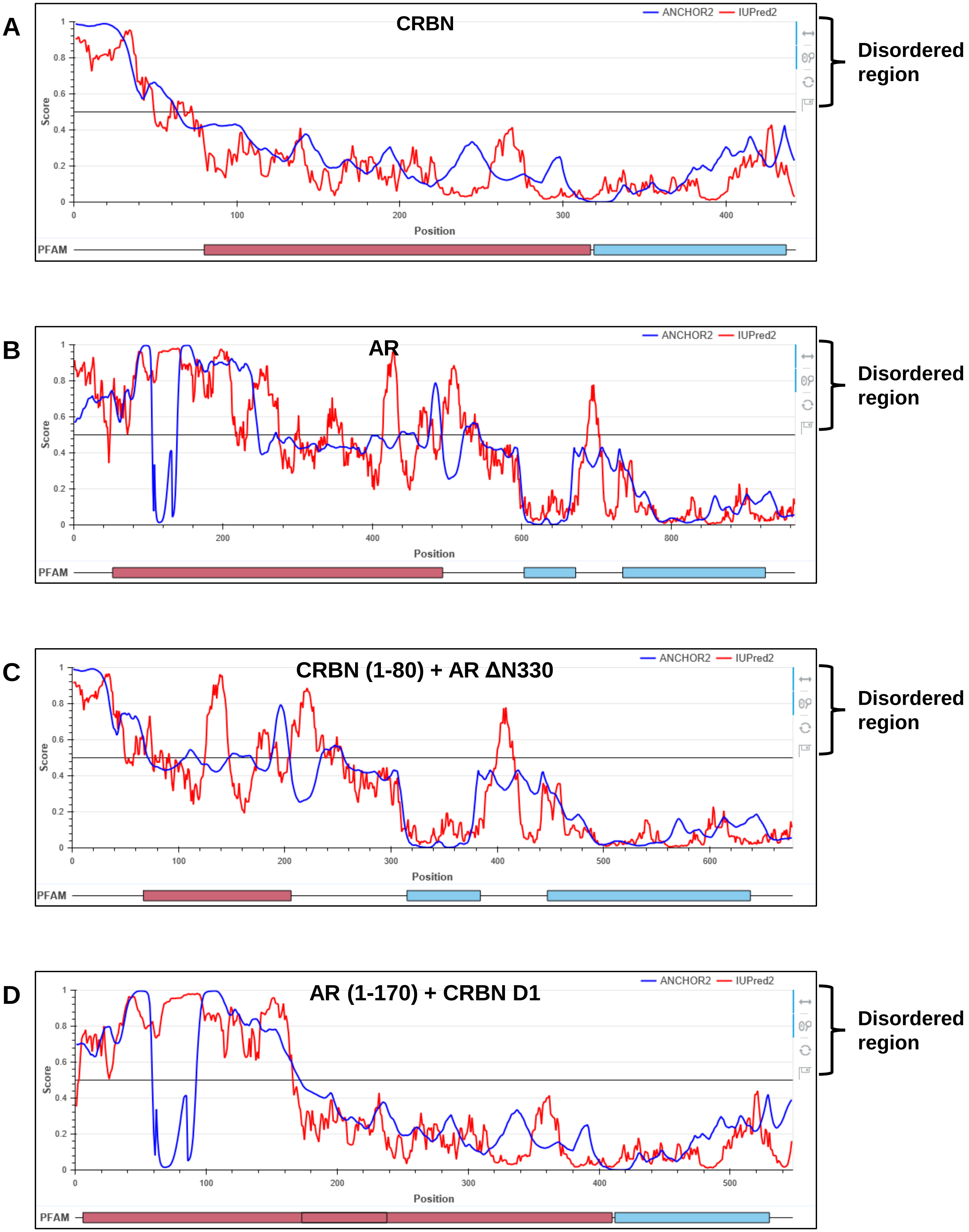

Supplementary Figure S5.

(A, B, C, D) Prediction of intrinsically unstructured regions in (A) CRBN, (B) AR, (C) CRBN (1-80 a.a) + AR ΔN330, and (D) AR (1-170 a.a) + CRBN D1 using IUPred2A

Supplementary Figure S6.

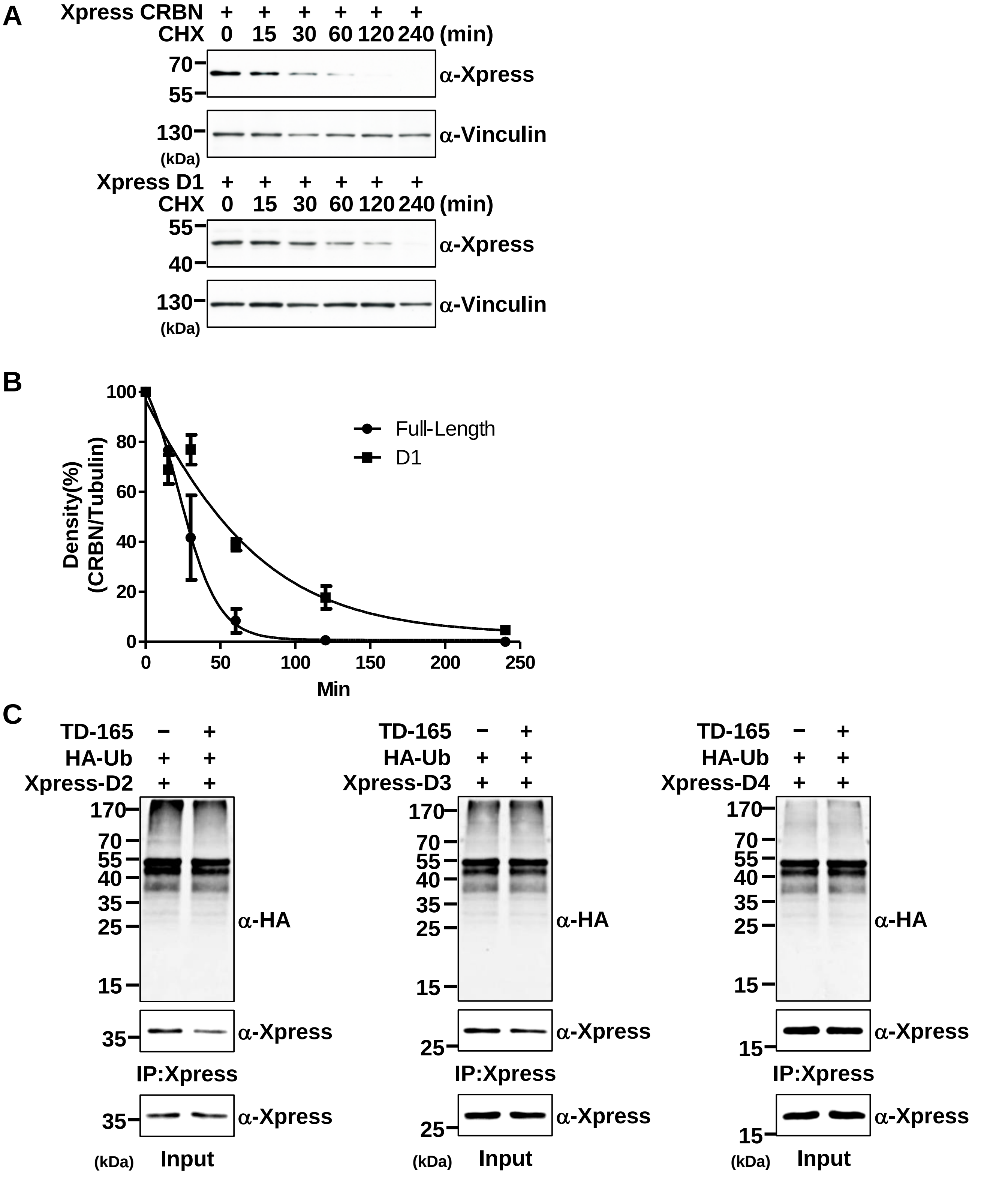

Supplementary Figure S6.

(A) Plasmids expressing full-lentgh CRBN and D1 were transfected into the HEK293T cells. After 8 h, the cells were harvested and divided into six groups. Each group was then treated cycloheximide (100 μg/ml) at different time points. Whole-cell lysates were analyzed by immunoblotting for the indicated proteins.. (B) Three independent experiments were quantified and drawn into graph. (C) Plasmids expressing Xpress tagged CRBN D2, D3 or D4 and HA tagged Ubiquitin were transfected into the HEK293T cells. After 36 h, the cells were treated with TD-165 (3 μM) and bortezomib (20 nM) or DMSO and bortezomib (20 nM) for 12 h. Whole cell lysates and the immunoprecipitated proteins using Xpress antibody were analyzed by immunoblot analysis for the indicated proteins.

Figure 1C and 1E full blots (Films)

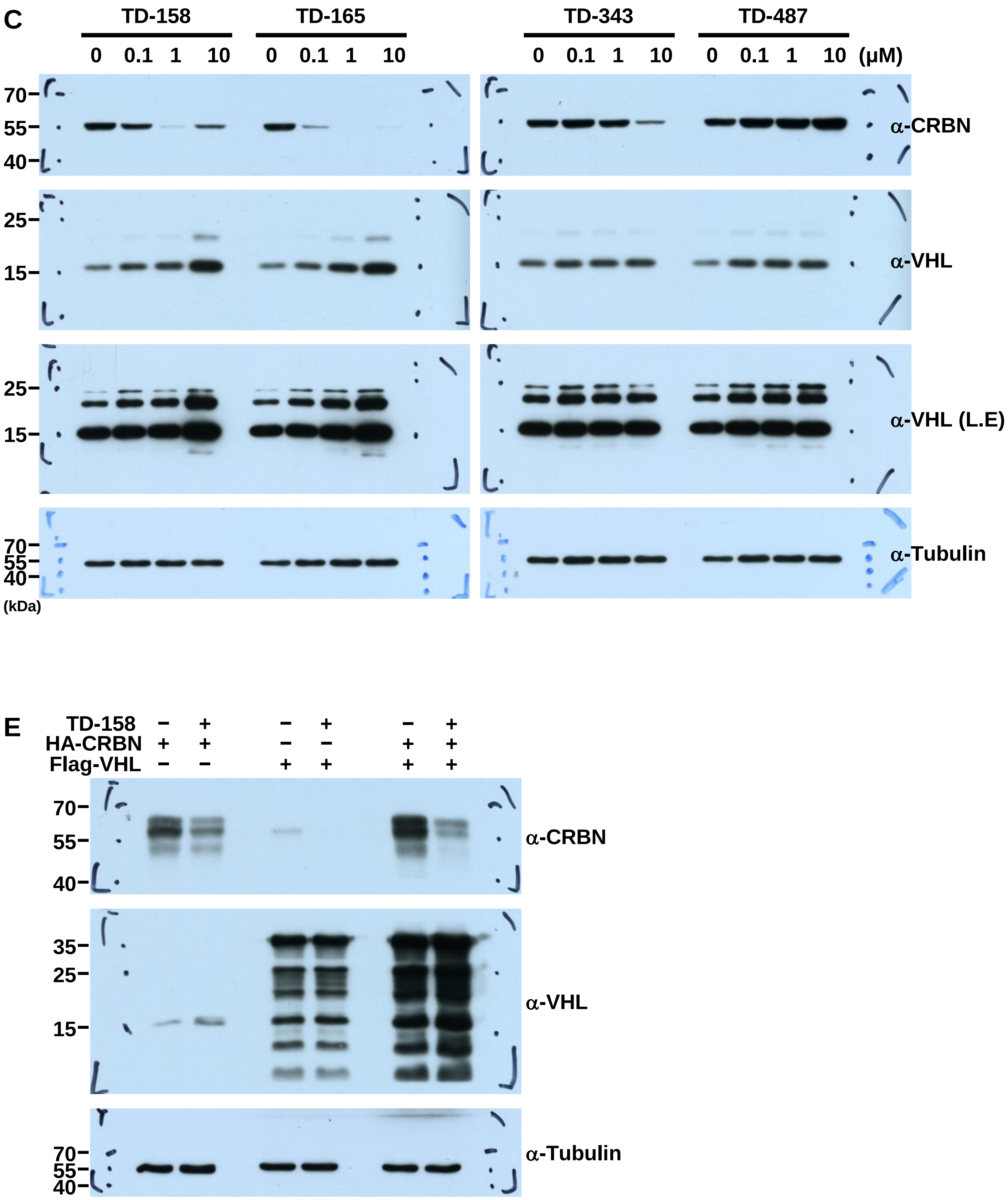

Figure 1F full blots (Films)

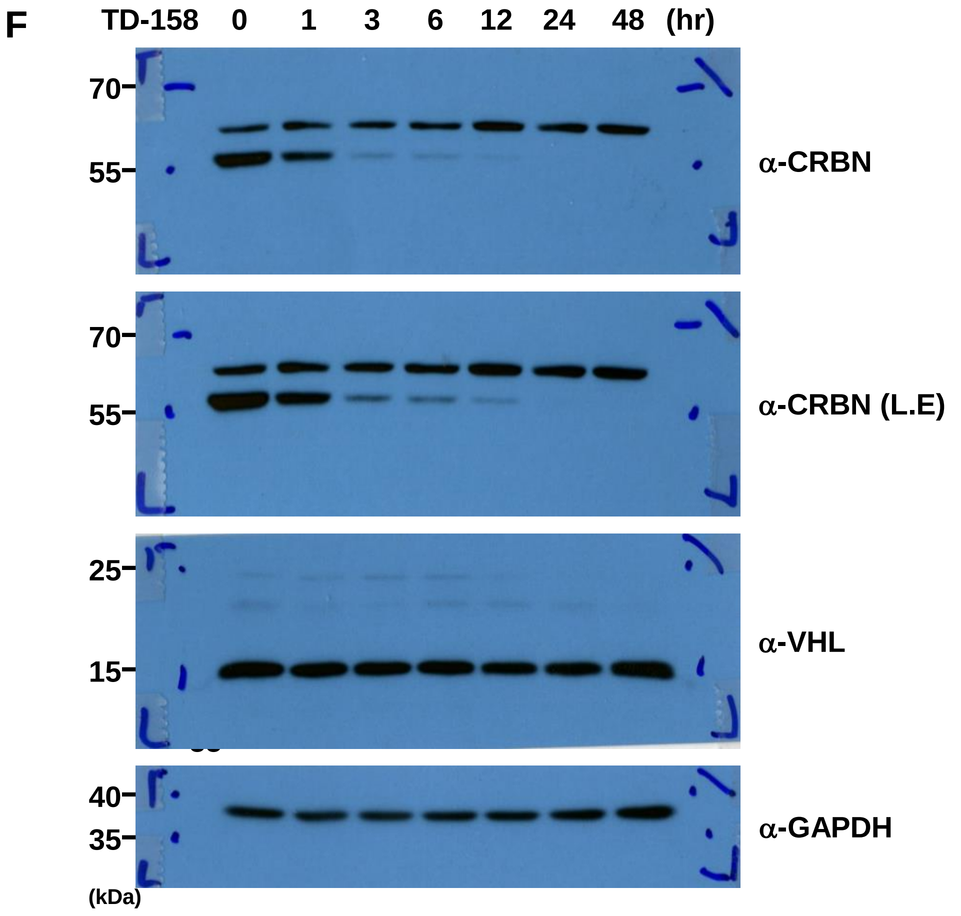

Figure 2A full blots (Amersham Imager)

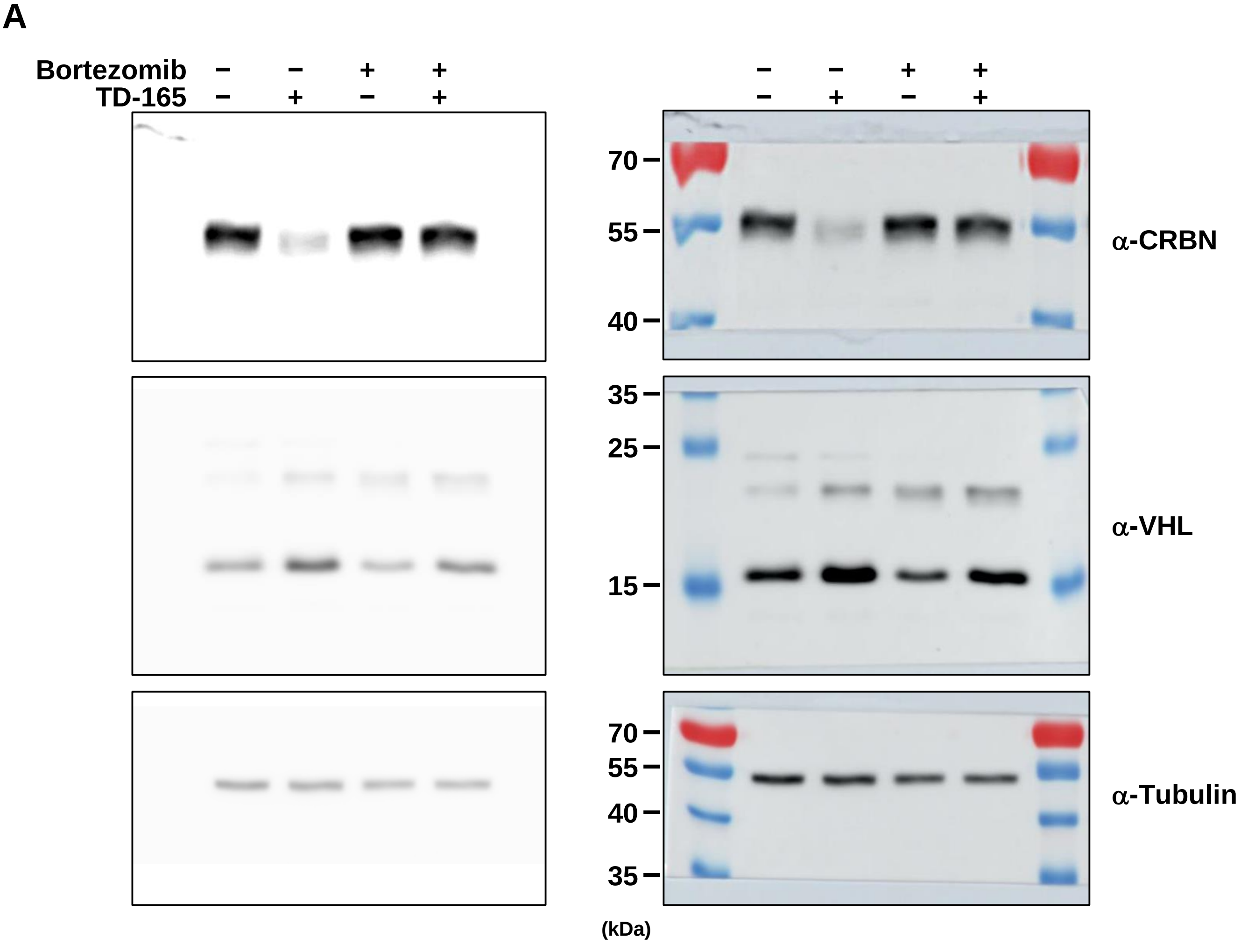

Figure 2B and 2C full blots (Films)

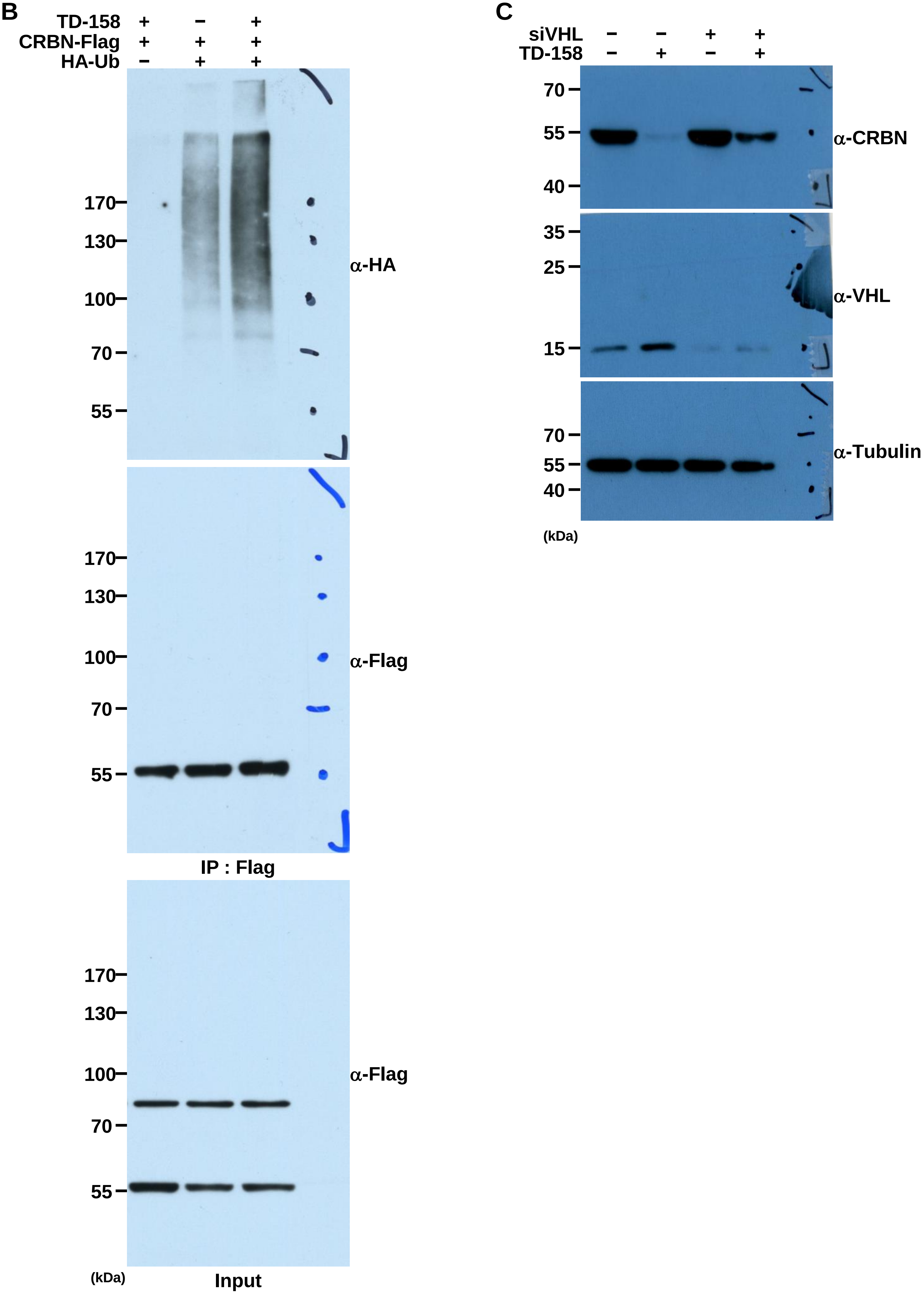

Figure 2D, 2E, 2F and 2G full blots (Films)

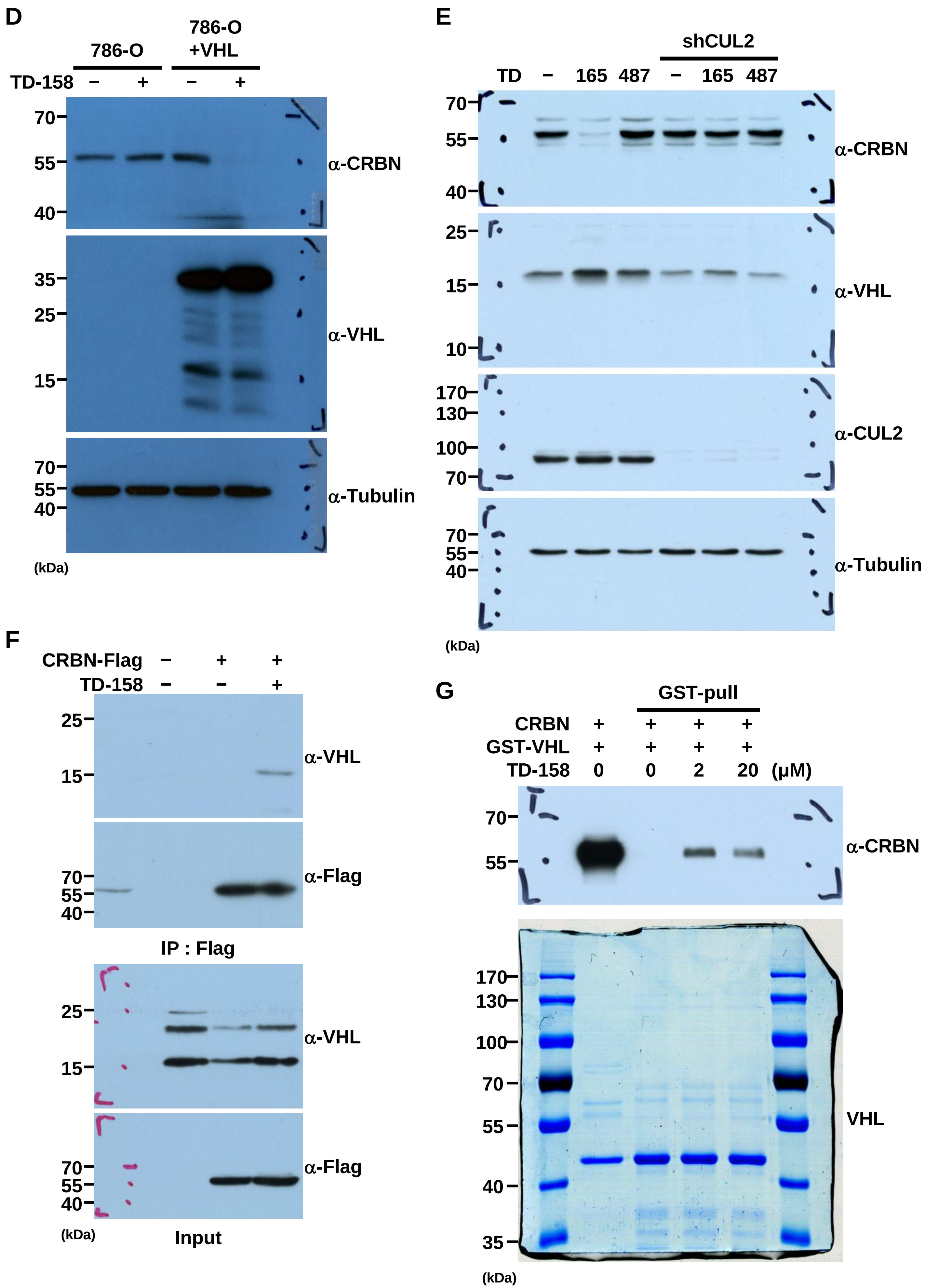

Figure 3C and 3D full blots (Films)

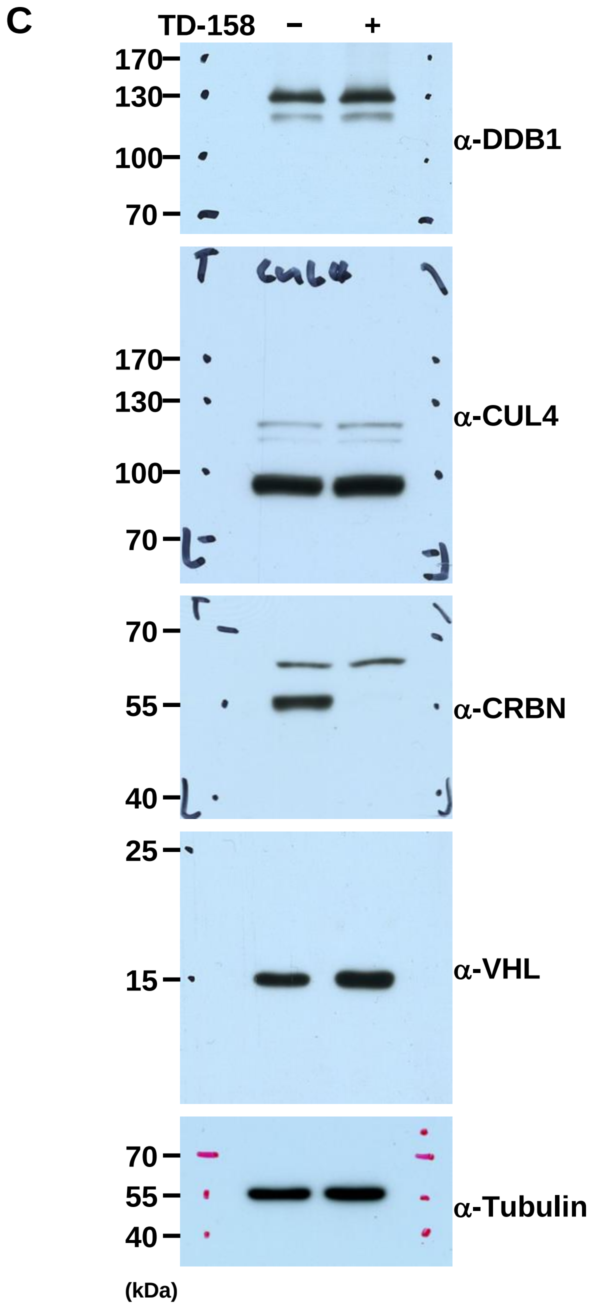

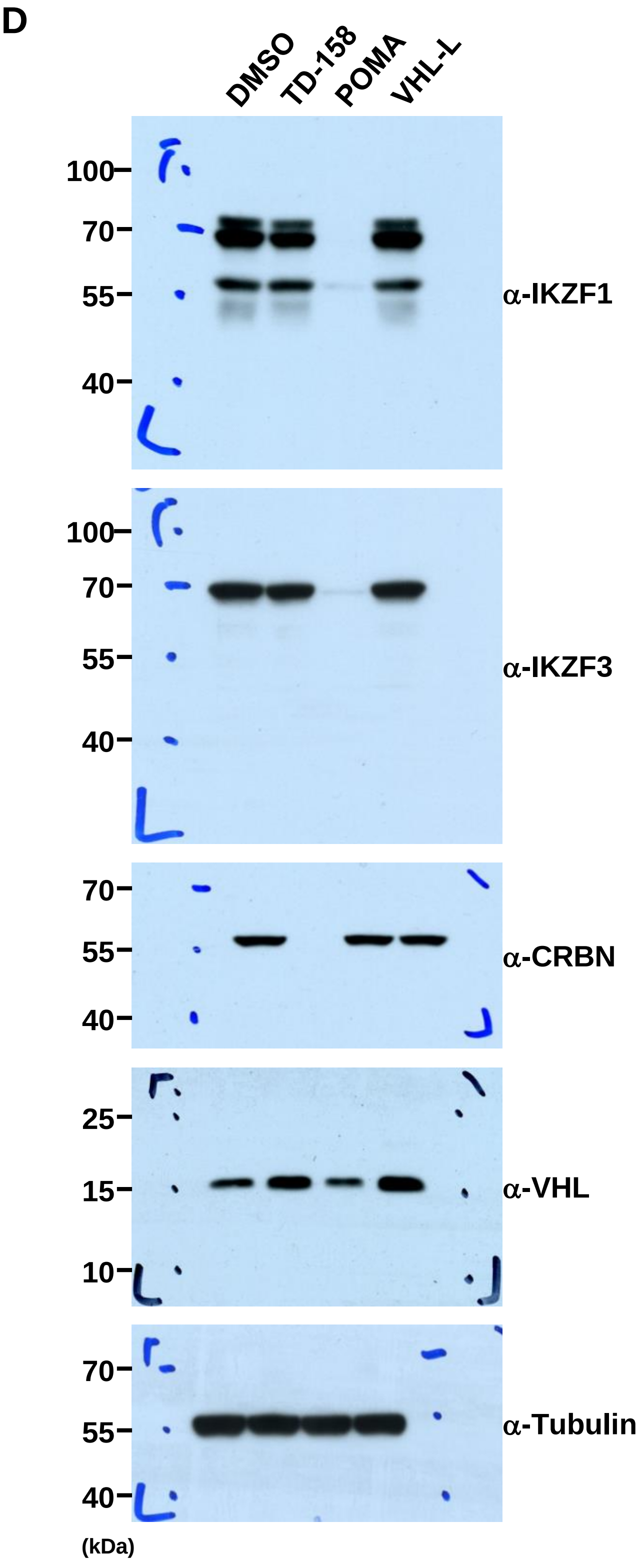

Figure 4A and 4C full blots (Films)

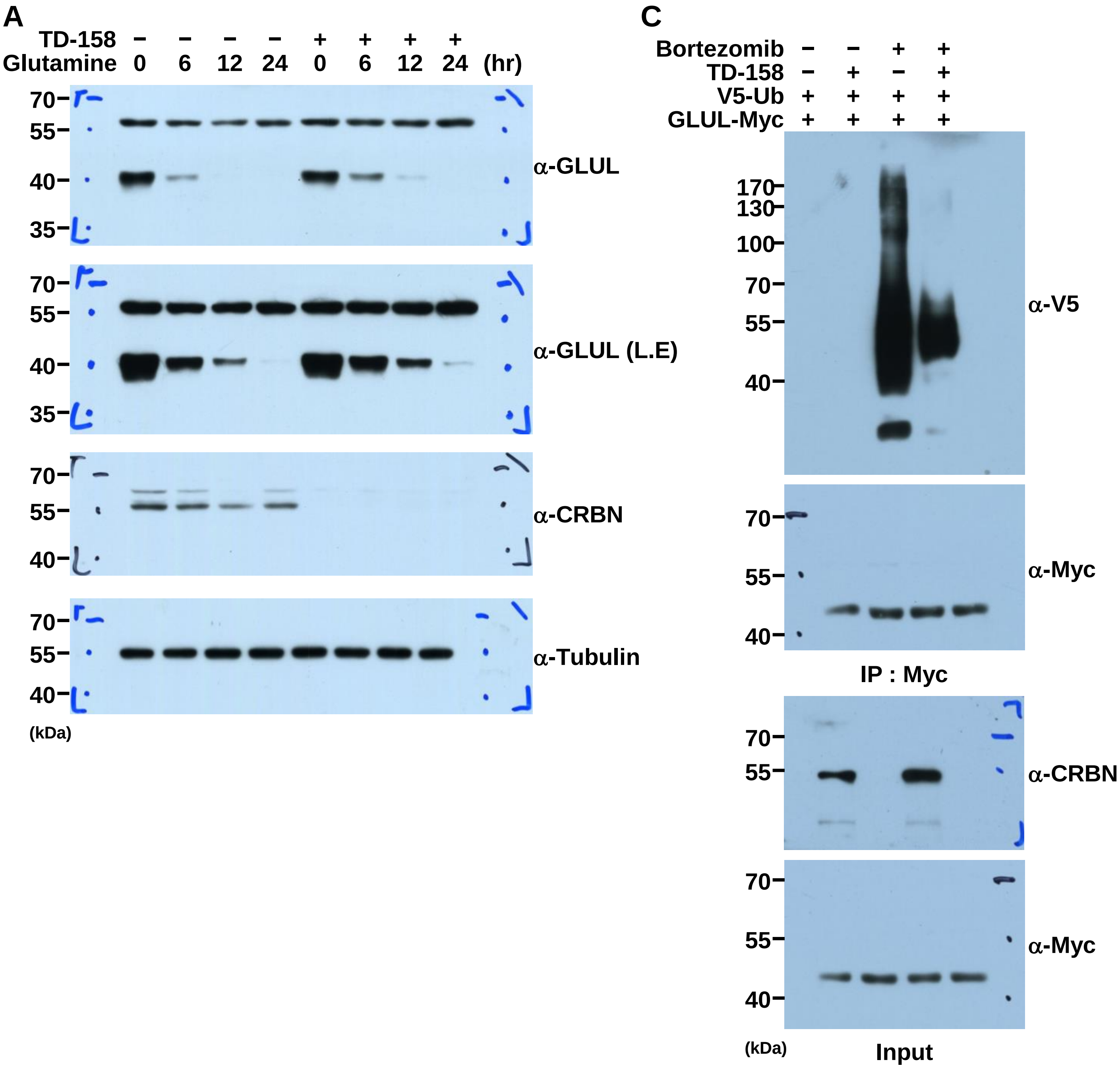

Figure 5B and 5D full blots (Amersham Imager)

B

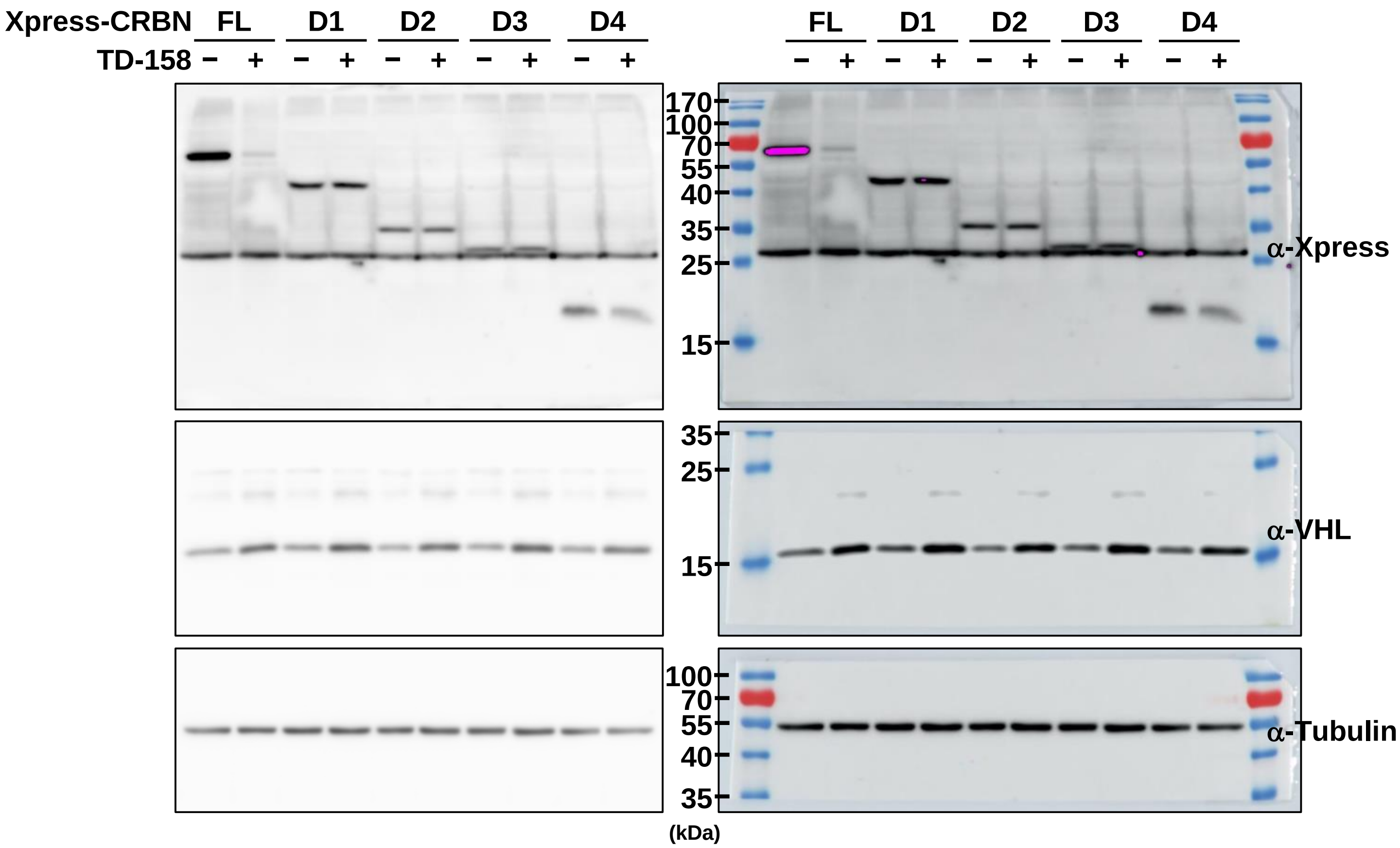

D

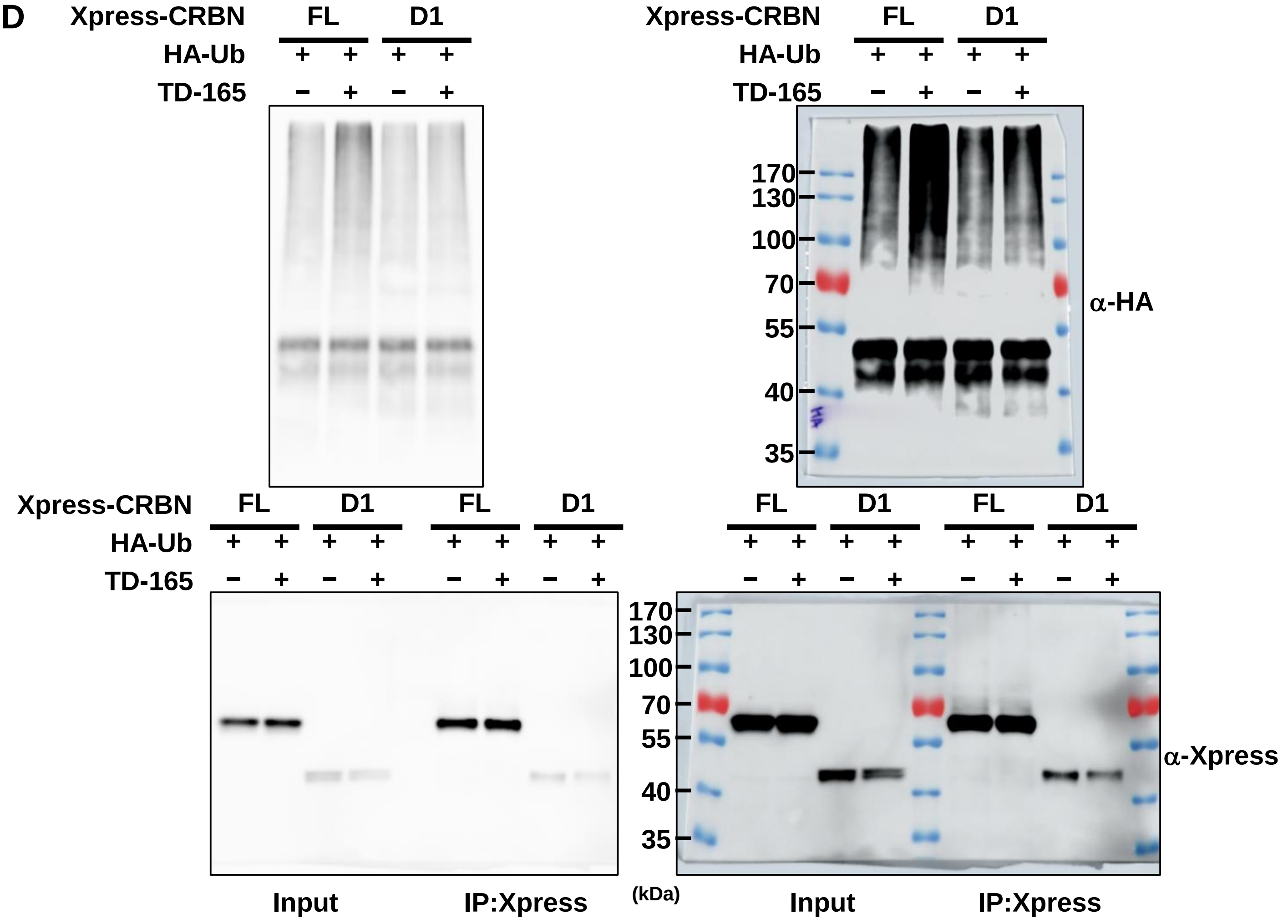

Figure 5C and 5E full blots (Films)

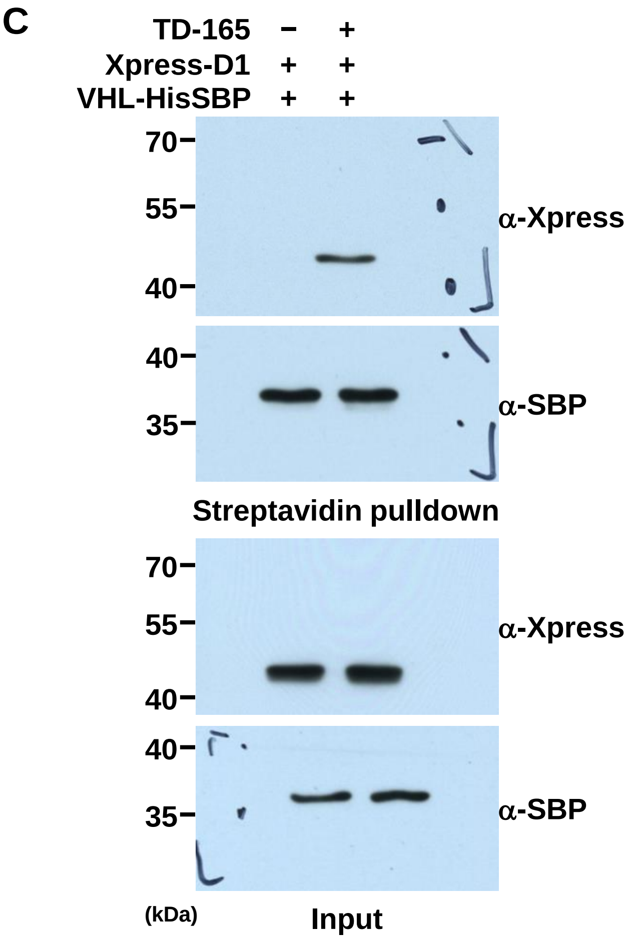

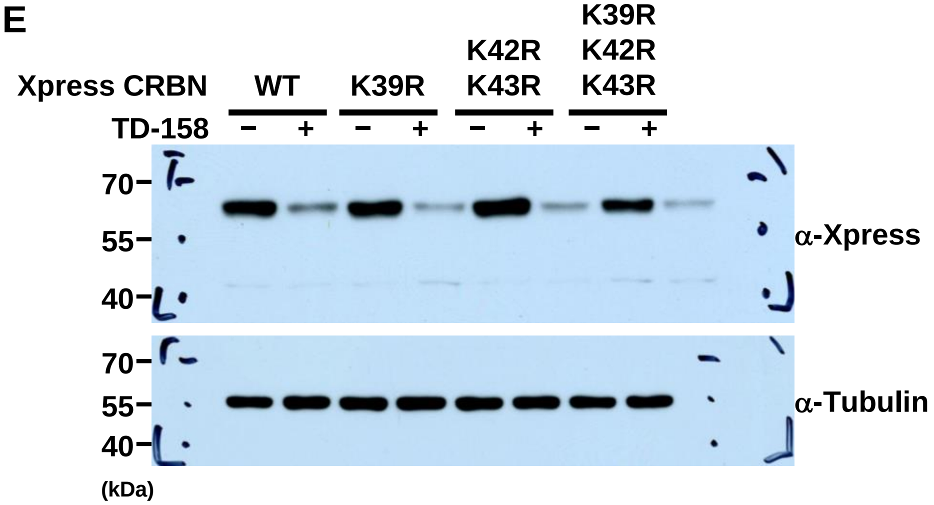

Figure 6A full blots (Amersham Imager)

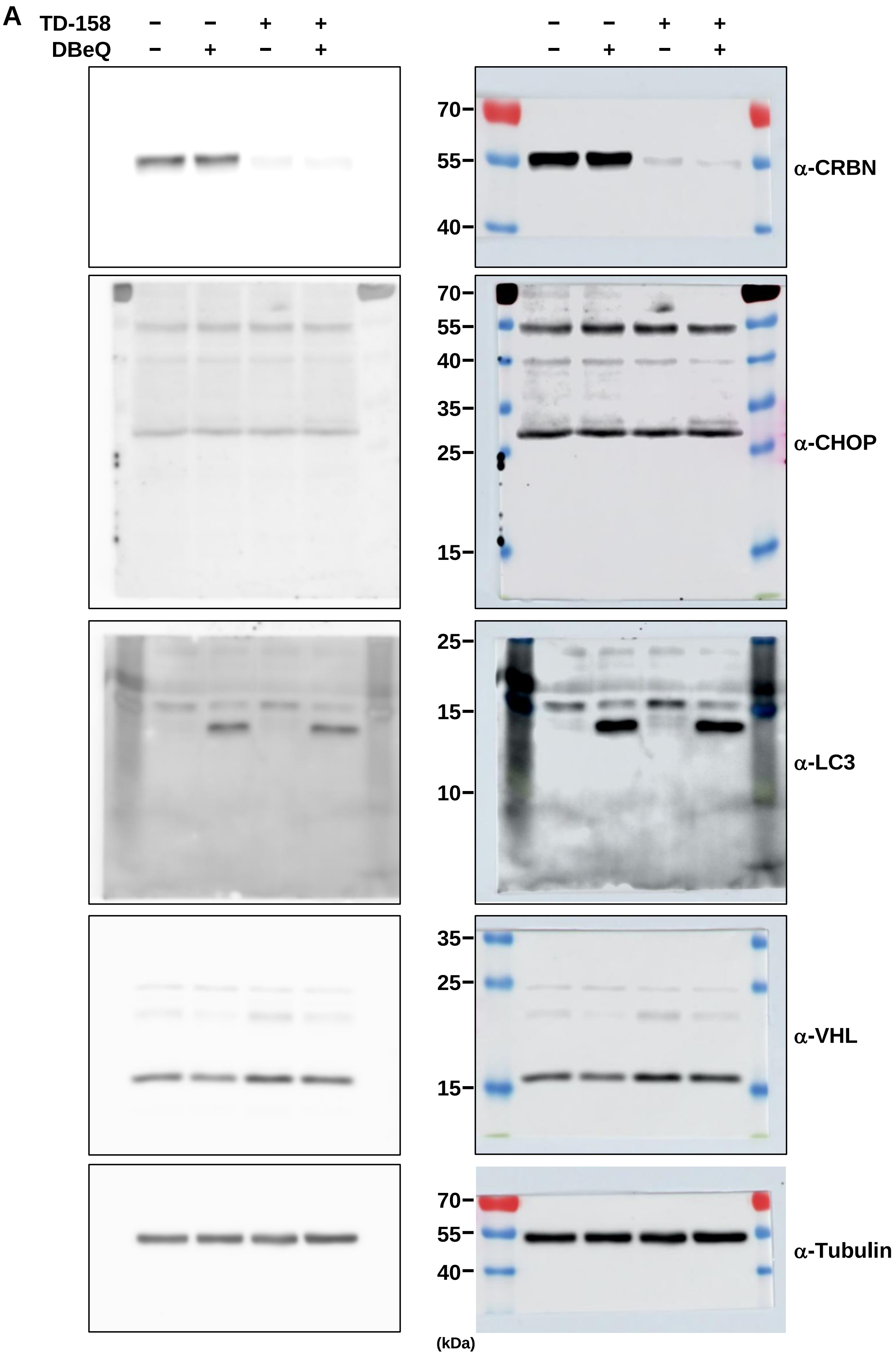

Figure 6B and 6C full blots (Films)

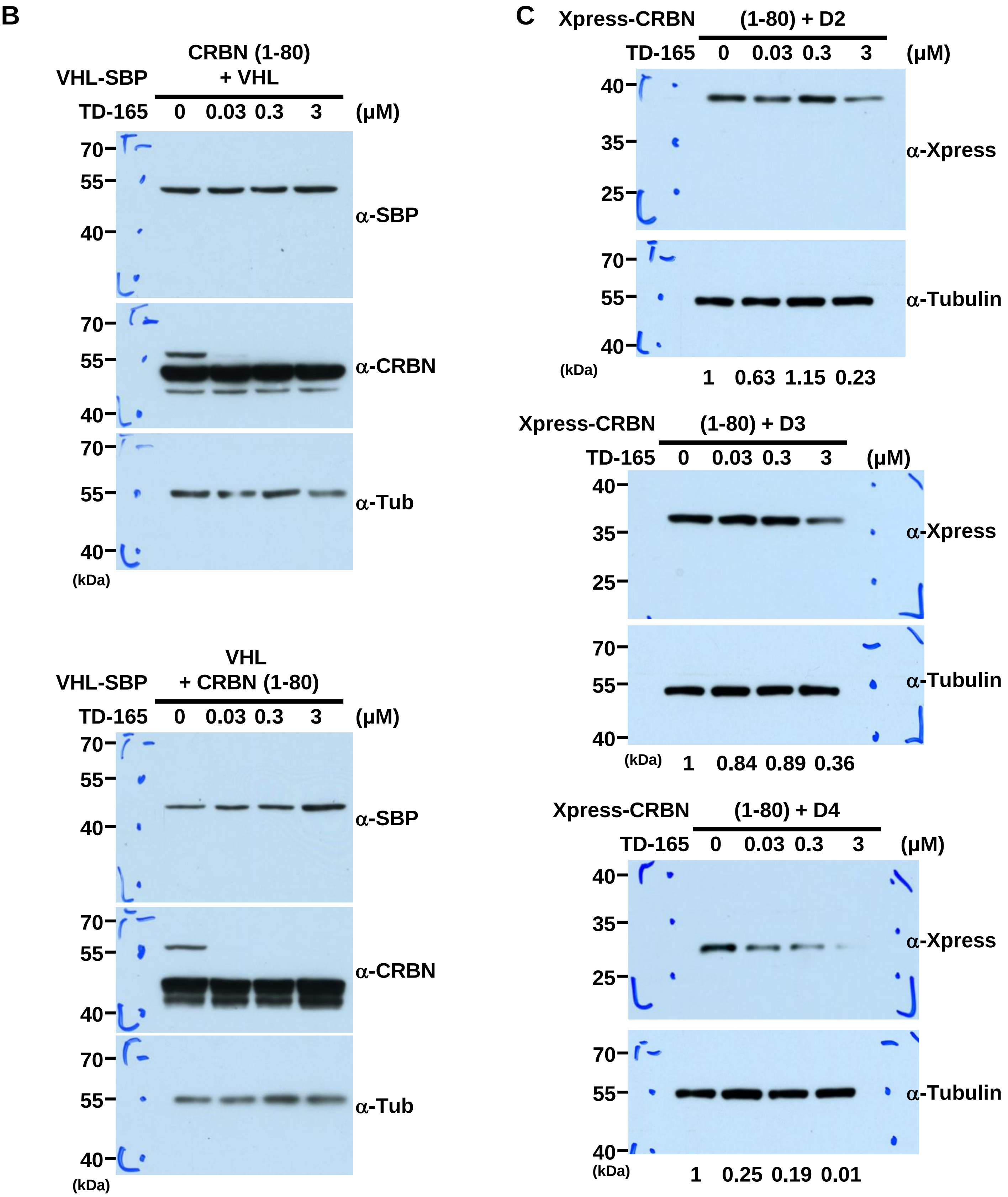

Figure 6D full blots (Amersham Imager)

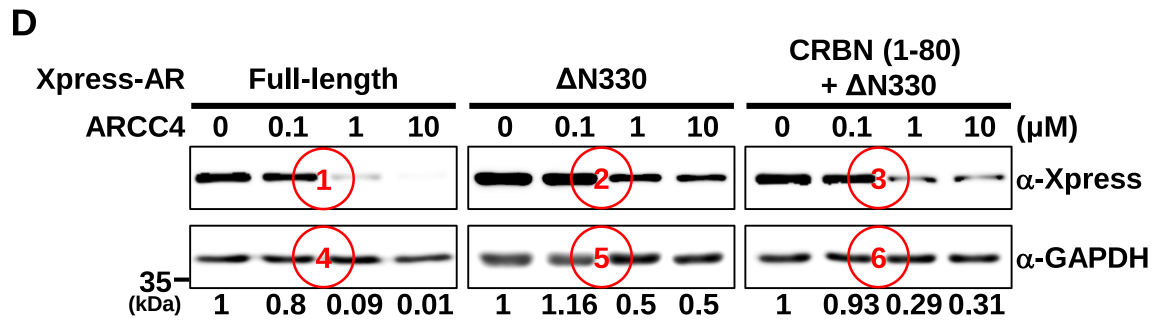

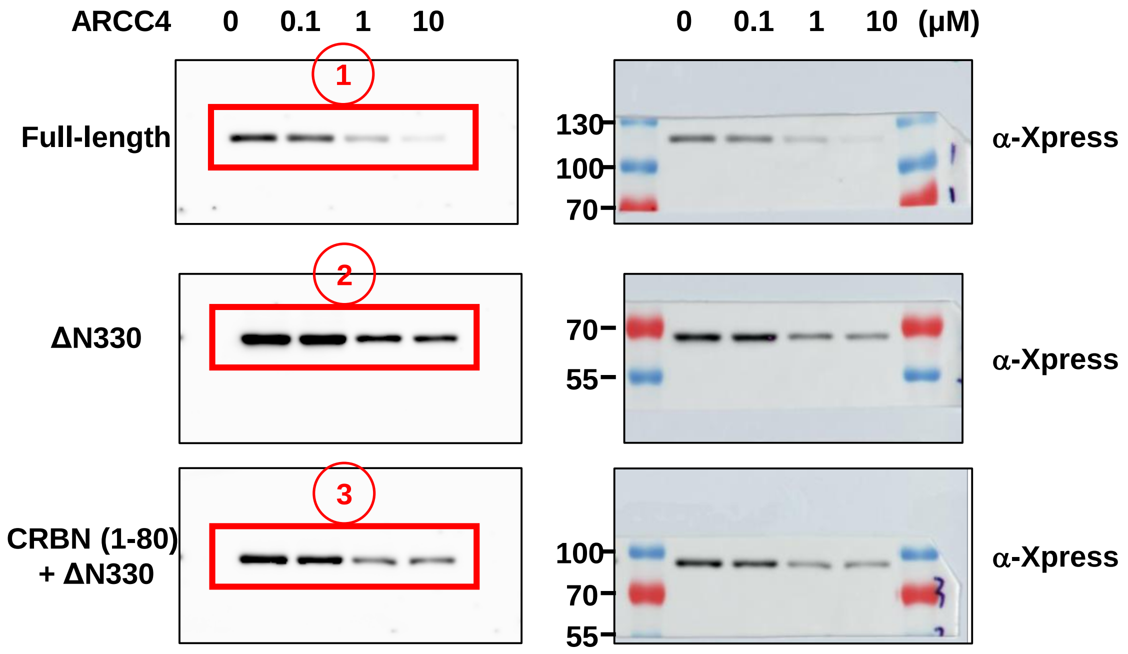

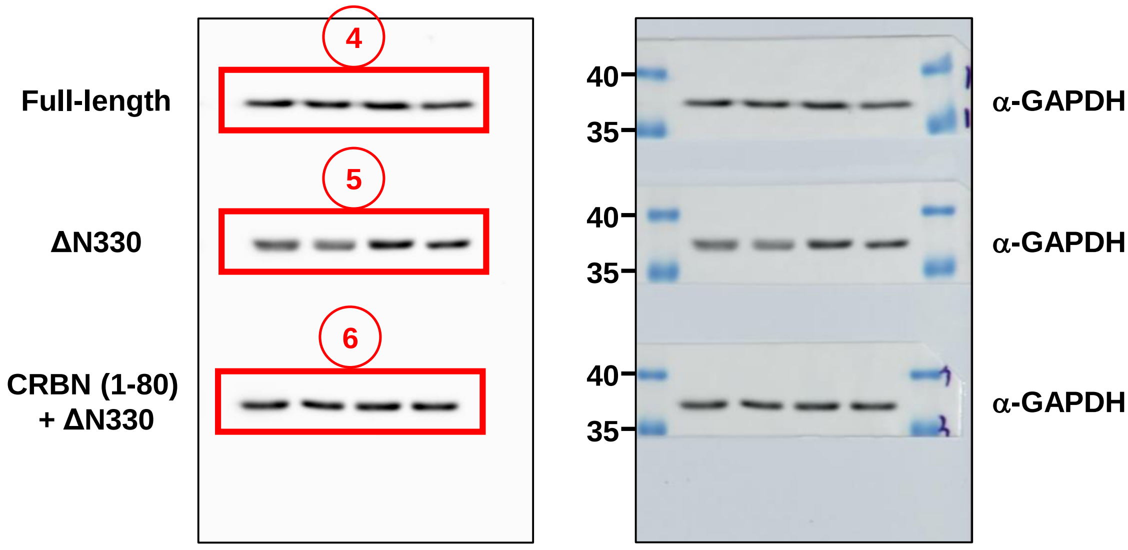

Figure 6E full blots (Films)

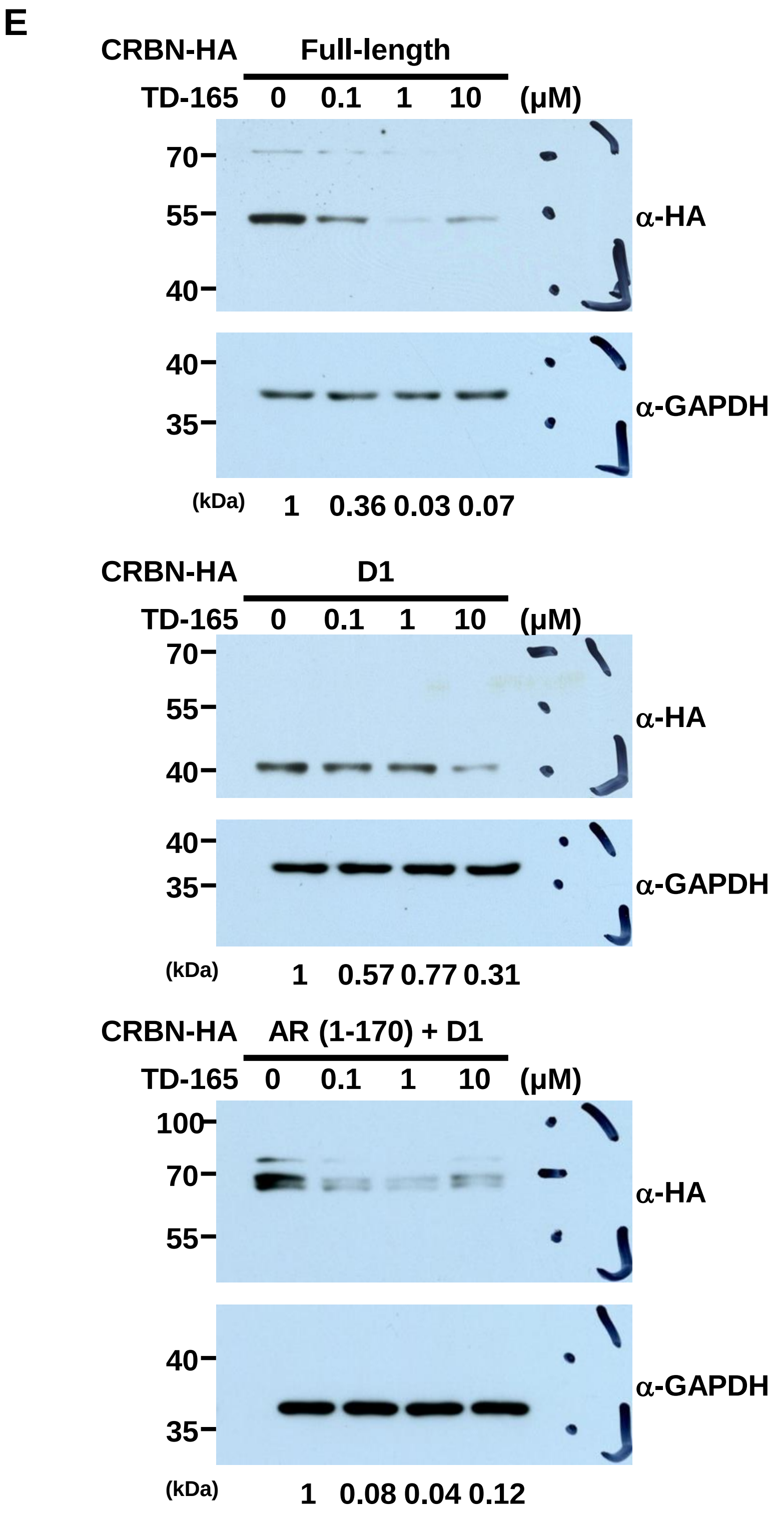

Supplement: Supplementary file 1 — supplementary information [file 41598_2019_56177_MOESM1_ESM.pdf]
